# Pathways, predictors and paradoxes of illbeing and wellbeing in older adults: Insights from a UK Biobank study

**DOI:** 10.1371/journal.pmen.0000336

**Published:** 2025-09-03

**Authors:** Tom C. Gordon, Andrew H. Kemp, Darren J. Edwards

**Affiliations:** 1 Department of Public Health, Faculty of Medicine, Health and Life Science, Swansea University, Swansea, United Kingdom; 2 School of Psychology, Faculty of Medicine, Health and Life Science, Swansea University, Swansea, United Kingdom; All India Institute of Medical Sciences, INDIA

## Abstract

This study presents the first UK Biobank analysis to concurrently model subjective wellbeing and illbeing within a unified biopsychosocial framework, offering a novel, data-rich perspective on psychological functioning in later life. While wellbeing and illbeing are often studied in isolation, there is growing recognition that their determinants may differ in kind and form. We address this gap by examining how biological, psychological, and social factors dynamically shape both outcomes in a large community-dwelling sample. Drawing on data from 8,047 participants (mean age = 64.8 years; 46.7% male; 90.7% White British), we constructed a theory-informed partial least squares structural equation model (PLS-SEM) linking heart rate variability (HRV), meaning-oriented behaviour (MOB), resilience, social connectedness, and lifetime adversity to wellbeing and illbeing. Model robustness was supported through 10,000-sample bootstrapping and split-half replication. Network centrality analysis (NCA) was used to identify key drivers, and Bayesian regression was applied to test non-linear functional forms for each path, validated using a held-out test dataset. MOB emerged as the strongest direct predictor of both increased wellbeing and reduced illbeing. HRV influenced wellbeing indirectly via psychosocial mediators. Adversity had the largest total effect on illbeing but no direct effect on wellbeing. Together, predictors accounted for ~52% of variance in both outcomes. Bayesian models revealed exponential, cubic, and logarithmic forms, indicating that conditions optimising wellbeing are not merely the inverse of those reducing illbeing. These findings offer a detailed mapping of non-linear biopsychosocial pathways in older adults and challenge the assumption that wellbeing and illbeing lie on a single continuum. The study provides a robust empirical foundation for developing process-based, context-sensitive mental health interventions. Longitudinal and more demographically diverse studies are now needed to test causal directions and broader generalisability.

## Introduction

Understanding the dynamic interplay between wellbeing and illbeing is essential for refining psychological theory and informing clinical practice. Wellbeing has traditionally been conceptualised through hedonic (pleasure-based) and eudaimonic (meaning-based) perspectives [1], and recent third-wave therapies (including Acceptance and Commitment Therapy or ACT, and positive psychotherapy) emphasise meaning-oriented behaviour (MOB), psychological flexibility, and vagal function (indexed by heart rate variability; HRV) as key determinants of wellbeing [2,3]. Illbeing, by contrast, encompasses psychological and physical symptoms such as anxiety, depression, and poor health, which contribute substantially to global disease burdens and mortality [4]. These two constructs are often assumed to lie on a single continuum, with wellbeing representing the absence of illbeing [5]. However, evidence indicates that individuals can experience both simultaneously [6,7]. This points to a more complex, multidimensional relationship, where the pathways leading to wellbeing and illbeing may be overlapping yet distinct, and potentially non-linear [8]. This study investigates such complexity using a large UK Biobank sample [9], guided by third-wave frameworks that focus on meaning, flexibility, and biopsychosocial integration [2,3,10]. Specifically, we examine: (1) whether wellbeing and illbeing are empirically separable and functionally distinct; (2) how MOB and vagal function shape these outcomes; and (3) the mediating or moderating roles of adversity, social connectedness, and resilience.

Contemporary therapeutic models such as process-based therapy (PBT) shift the focus from categorical diagnoses to processes of change [11]. They are informed by frameworks like the Extended Evolutionary Meta Model (EEMM) [12] and the GENIAL model [3,13], which integrate psychological, physiological, and social dimensions of mental health. Vagal function, indexed by HRV, reflects the body’s capacity for stress regulation and has been associated with emotional stability, social engagement, and overall wellbeing [14,15]. However, findings are inconsistent [16], suggesting the presence of upstream moderators and complex, possibly non-linear relationships [3,8]. Similarly, personal meaning systems characterised by value coherence and differentiation predict mental and physical health outcomes [17,18]. ACT and positive psychotherapy have been shown to increase HRV indirectly through promoting behaviours aligned with personal values, emotional regulation, and psychological acceptance [10,19–21]. Thus, both HRV and MOB may act as upstream drivers of wellbeing, yet their pathways remain underexamined.

Resilience (the capacity to adapt positively to adversity), is also a central construct [22]. Among those with chronic conditions, resilience and personal growth are bolstered by a sense of meaning and supportive relationships [23,24]. Third-wave therapies promote resilience through strategies such as acceptance, defusion, and values-based action [25], and the GENIAL framework emphasises its connection with emotion regulation, social bonds, and environmental connection [3,10,26]. Structural equation modelling (SEM) research supports these links, showing resilience to be associated with higher wellbeing and reduced impact of stress [27,28]. Social connectedness is similarly a robust predictor of both enhanced wellbeing and reduced illbeing, shown in SEM research to have a mutually beneficial association with perceived meaning in life [29]. It mediates the effects of stigma and isolation in chronic illness and is linked to quality of life across psychological and physical domains [30]. Other SEM studies reinforce its positive associations with health behaviours, subjective wellbeing, and lower psychological distress, as well as a role in mediating the effects of gratitude on wellbeing [28,31], aligning with theories suggesting that social connection is a fundamental psychological need [12,13,32,33].

Traditional models often assume linear relationships between wellbeing and illbeing [8,34], yet growing evidence suggests otherwise [4,35]. Individuals can experience high life satisfaction alongside psychological distress [36]. This has led to the dual-continua model, which posits that wellbeing and illbeing are separable dimensions [6]. Earlier versions delineated three states, including flourishing (high wellbeing, low mental illness), languishing (low wellbeing, low mental illness), and struggling (low wellbeing, high mental illness), whereas recent extensions of the model include a “symptomatic but content” category, recognising that high wellbeing can coexist with high distress [37]. Such findings highlight the need to foster meaning and positive functioning alongside efforts to reduce symptoms [37–39].

Assuming linearity may obscure meaningful effects. For example, both low and high BMI are linked to poorer mental health, whereas moderate BMI is associated with better outcomes [8]. Similarly, moderate stress is associated with higher wellbeing and cognitive functioning, whereas both very low and very high levels of stress relate to poorer outcomes [40,41], a pattern consistent Yerkes-Dodson law (an inverted U-shaped relationship between performance and anxiety) [42]. Even basic predictors such as social connectedness may follow non-linear patterns, for example introverts may thrive with lower levels of social contact, whereas extroverts might benefit from greater engagement [43]. Over-reliance on linear models risk overlooking such context-sensitive dynamics, inflating false-positive rates and mischaracterising effect sizes [44–46]. Recognising quadratic or other non-linear functional relationships may help to inform personalised wellbeing strategies, but this remains under-explored.

Building upon these insights, the present study employs Partial Least Squares Structural Equation Modelling (PLS-SEM) to examine how MOB and vagal function might act as upstream drivers of wellbeing and illbeing, and how resilience, social connectedness and adversity may mediate or moderate these pathways. We also apply Bayesian regression to test potential non-linear relationships among the variables, clarifying how different levels of each factor my shape individual trajectories [47].

Research questions are as follows:

1. To what extent are subjective wellbeing and illbeing distinct constructs, and what is the functional nature of their relationship? What function best characterises the direct relationships between predictors (HRV, MOB, resilience, social connectedness, adversity) and outcomes (wellbeing, illbeing)?
2. How do MOB and vagal function influence the pathways leading to wellbeing and illbeing, and are these pathways distinctive?
3. What are the mediating and/or moderating effects of adversity, social connectedness, and resilience on these pathways?

Predictions for this study are:

1. The relationship between wellbeing and illbeing will be associated with considerable non-linearity. Predictors will vary in whether they have a linear, quadratic, sigmoid, cubic, logarithmic, or exponential relationship with illbeing or wellbeing.
2. MOB and vagal function will positively influence pathways to wellbeing and serve as buffers against pathways leading to illbeing.
3. The effects of MOB and vagal function on wellbeing and illbeing will be moderated by factors such as adversity and the quality of social connection, highlighting the importance of contextual and individual differences in psychological processes.

By integrating theoretical perspectives and empirical findings, this study aims to provide a comprehensive framework for better understanding the complex dynamics between wellbeing and illbeing.

## Methods

### Participants

We analysed data from 8,047 UK Biobank (UKBB) participants [9] who had completed every questionnaire required for the present study and the baseline resting-ECG data protocol in the same sitting (see Table 1). The UK Biobank, initiated in 2006, is a comprehensive longitudinal database focused on genetic and non-genetic predispositions to disease in adults aged 37–73 years. Participants were recruited from NHS records across the UK, with baseline data collected at 22 assessment centres between 2006 and 2010. The data for this study were accessed on 15 April 2024. The UK Biobank obtained ethical approval from the Northwest Multicentre Research Ethics Committee, and all participants provided written informed consent (ref: 16/NW/0274; see: https://www.ukbiobank.ac.uk/learn-more-about-uk-biobank/about-us/ethics). The dataset is fully anonymised, and the authors had no access to identifying information. Relevant approval for the current study was also granted by the UK Biobank Data Access Committee (Approved Research ID: 92623).

**Table 1:** Participant demographics.

	Total sample	Male (n = 3,760; 46.74%)	Female (n = 4,288; 53.26%)
Age, years: mean (s.d.)	64.79 (7.74)	65.73 (7.78)	63.98 (7.60)
White British n (%)	7296 (90.67%)	3460 (92.00%)	3836 (89.50%)
White Other n (%)	449 (5.58%)	178 (4.73%)	271 (6.32%)
South Asian n (%)	95 (1.18%)	57 (1.52%)	38 (0.89%)
Black Groups n (%)	55 (0.68%)	17 (0.45%)	38 (0.89%)
Other n (%)	152 (1.89%)	49 (1.30%)	103 (2.40%)

### Patient and public involvement

Patients and the public were not involved in the design, conduct, reporting, or dissemination plans of this research.

### Analytical Approach

A two-phase analytical strategy, exploratory followed by confirmatory, was adopted to map the pathways to, and the relationships between, subjective wellbeing and illbeing, following the STROBE (Strengthening the Reporting of Observational Studies in Epidemiology) checklist to ensure transparent and comprehensive reporting [48] (see S1 Checklist). Because sex and gender were not primary research questions, data were not disaggregated on these variables. The aim was to characterise broader psychosocial pathways irrespective of these differences.

In the exploratory phase, PLS-SEM was used to test relationships between key constructs specified in our conceptual model (see S1 File). PLS-SEM was selected for its capacity to accommodate complex latent-variable structures, tolerate non-normal data, and maximise explained variance in outcomes [49]. Confirmatory analyses included bootstrapping and split-half validation to assess the stability and reliability of findings. Bootstrapping involved generating 10,000 resamples to derive confidence intervals and significance estimates. Split-half validation was performed by randomly dividing the sample and repeating the analysis in each subset to assess the consistency of model paths and explained variance [50]. Although PLS-SEM estimates directional relationships based on theoretical specification, the resulting path coefficients reflect conditional associations derived from cross-sectional data. As such, they may be consistent with causal processes but cannot confirm them [51].

To complement the PLS-SEM, Network Centrality Analysis was conducted using the resulting path coefficients to construct an adjacency matrix of latent constructs. Degree, closeness, and betweenness centrality were computed to capture different aspects of construct influence: number of direct connections, efficiency of information flow, and role as bridging variables, respectively. Functional transformations were then applied to test for non-linear patterns, followed by Bayesian regression modelling to assess the shape and strength of confirmed direct paths. This approach allowed flexible modelling of complex, potentially non-linear relationships between predictors and outcomes. For a detailed summary of each statistical method and its interpretation, see Table 2.

**Table 2:** Key statistical terms/parameters and explanations.

Key Statistical Terms	Explanation	Key Parameters & Interpretation
Partial Least Squares Structural Equation Modelling (PLS-SEM)	A statistical approach used to model complex relationships between observed variables and underlying latent constructs. PLS-SEM is well suited for exploratory research, especially when working with many constructs, small effect sizes, or non-normally distributed data. Unlike traditional SEM, which focuses on reproducing the full covariance matrix, PLS-SEM prioritises maximising the explained variance (R²) in key outcomes. It also handles both reflective and formative measurement models, and is particularly effective for identifying indirect effects and prediction pathways in large, theory-driven models.	**Indicator Loadings:** Values ≥0.70 indicate strong contributions of observed variables to a latent construct. **Internal Consistency Reliability:** Cronbach’s alpha and composite reliability >0.70 suggest strong consistency among indicators. **Convergent Validity:** Average Variance Extracted (AVE) >0.50 means over half the variance in indicators is accounted for by the latent construct. **Discriminant Validity:** Heterotrait–Monotrait Ratio of Correlations (HTMT) <0.85 supports the distinctiveness of constructs; higher values suggest overlap. **Predictive Relevance:** Q² values >0 indicate that the model predicts the target construct better than chance.
Bootstrapping	A resampling method used in PLS-SEM to assess the stability and significance of model estimates. It constructs empirical sampling distributions by repeatedly drawing samples (with replacement) from the original data, allowing inference without assuming normality. Bootstrap output is used to assess significance, evaluate fit indices, and validate model robustness.	**Number of Resamples:** Typically 5,000–10,000. Larger resamples improve the precision of estimates and stability of confidence intervals. **Significance Testing:** Path coefficients are evaluated using t-values, p-values, and bias-corrected confidence intervals derived from the bootstrap distribution. This includes indirect and interaction effects, which lack standard error formulas. **Model Fit Indices:** The Standardised Root Mean Square Residual (SRMR) <0.08 indicates good fit. The Normed Fit Index (NFI) compares model fit to a null model; values >0.90 are desirable in simpler models. **Split-Half Cross-Validation:** The dataset is randomly divided in half. The model is estimated separately in each subset using bootstrapping. Consistency in path coefficients and explained variance across splits supports model robustness and guards against overfitting.
Network Centrality Analysis (NCA)	A method used to identify the most influential variables (or nodes) in a network by analysing the pattern of connections between them. Centrality metrics highlight which constructs have the most direct effects, serve as key intermediaries, or efficiently influence others. In this study, NCA was applied to the structural network defined by PLS-SEM path coefficients, allowing us to visualise and quantify the relative importance of each latent construct in shaping wellbeing and illbeing.	**Degree Centrality:** Counts the number of direct connections (immediate influence). **Closeness Centrality:** Inverse of the average shortest path length (efficiency in influence spread). **Betweenness Centrality:** Frequency with which a node lies on the shortest paths between others (bridge function).
Functional Transformations	Tests different mathematical forms to model the relationships between predictors and outcomes, allowing the detection of non-linear, threshold, or saturation effects. In this study, transformations were applied to the latent variable scores derived from the PLS-SEM, treating them as predictors and outcomes in a series of Bayesian regression models. For each predictor, multiple functional forms were compared to identify the best-fitting option.	**Function Interpretation:** • Linear: Constant rate of change. • Quadratic: Curved relationship (e.g., U-shaped or inverted U-shaped). • Cubic: Complex curve with multiple turning points. • Exponential: Rapid increases or decreases. • Logarithmic: Strong early effects that taper off. • Sigmoid: S-curve showing thresholds or saturation points. **Model Fit Evaluation:** Form selection was based on WAIC and LOO, which assess out-of-sample predictive performance while penalising overfitting. • WAIC (Widely Applicable Information Criterion): Estimates expected predictive accuracy; lower scores indicate better fit adjusted for model complexity. • LOO (Leave-One-Out Cross-Validation): Tests generalisability by predicting each observation from the rest. Lower values reflect stronger model performance across the dataset.
Bayesian Regression	A probabilistic modelling approach that estimates a full distribution of likely values (posterior distributions) for each parameter, based on prior beliefs and observed data. These distributions are approximated using Markov Chain Monte Carlo (MCMC), which simulates thousands of values to represent uncertainty. The model used the No-U-Turn Sampler (NUTS), an adaptive MCMC algorithm that efficiently explores the range of possible values by avoiding redundant or inefficient sampling paths. Multiple chains are run from different starting points to ensure the algorithm reaches the same conclusions regardless of where it begins, known as convergence, which helps confirm that the results are stable and reliable.	**Posterior Estimates (β, SD):** Mean reflects the average estimated effect; SD indicates uncertainty in the estimate. **Credible Intervals (HDI):** Highest Density Intervals show the most credible range of values for each parameter (e.g., 95% HDI). **Region of Practical Equivalence (ROPE):** Region around zero (e.g., ± 0.05) used to evaluate whether an effect is practically meaningful. If <5% of the posterior lies within the ROPE, the effect is considered non-negligible. **Convergence (R-hat):** R-hat ≈ 1.00 indicates the MCMC chains have converged on a stable posterior distribution. **Effective Sample Size (ESS):** Reflects the number of effectively independent samples; higher values (>1000) suggest stable estimates.
Bayesian Model Validation	Evaluates how well the Bayesian model generalises to new, unseen data. This guards against overfitting and supports conclusions about predictive performance. In this study, validation involved splitting the data into training and test sets, using the training data to estimate the model and the test data to evaluate out-of-sample predictions. Additional comparisons were made using WAIC and LOO, which assess how well different model specifications perform while accounting for complexity.	**Train-Test Split:** The dataset was randomly divided into training and test subsets. Model fit on the test set was used to assess predictive accuracy and generalisability. **Bayesian R²:** Proportion of variance in the outcome explained by the model. Higher values reflect stronger predictive fit. **MAE/ RMSE:** Mean Absolute Error and Root Mean Square Error evaluate how closely predictions match observed values. Lower scores indicate more accurate predictions. **WAIC & LOO:** Used to compare functional forms for each predictor. Lower values indicate better predictive fit while penalising complexity.

In conducting our analysis, we employed complete case analysis (CCA) due to the high proportion of missing data, which averaged 64.43% across key variables in the full biobank cohort (n > 500,000). Our inclusion of a comparatively large number of questionnaire items, along with physiological data, placed stricter demands on data completeness, ensuring a complete dataset for constructing robust latent variables in our theoretical model. Data from the UK Biobank cohort likely violates the assumption of Missing at Random (MAR), particularly in areas related to mental health measures, where missingness is often influenced by the underlying condition [52]. Given that multiple imputation (MI) relies heavily on the MAR assumption to produce unbiased estimates, it was deemed unsuitable for our dataset [53,54]. As supported by previous research, MI can introduce significant biases when a large portion of the data (>50%) is missing, especially when MAR cannot be assumed [55,56]. Consequently, CCA was determined to be the most robust approach, allowing us to focus on complete cases and thus ensure the validity of our findings [57]. Similar approaches have been adopted in other UK Biobank studies to maintain the reliability and robustness of the results when faced with significant missing data [52,58–60].

### Partial-least-squares Structural Equation Modelling (PLS-SEM)

PLS-SEM was employed on the full dataset (n = 8,047) using the SmartPLS 4.0 software [61], to investigate the relationships between subjective wellbeing, illbeing, and predictors including MOB, resilience, social connectedness, adversity, and HRV.

### Measurement model construction

Latent constructs were developed based on our conceptual model (see S1 File) and included both reflective and formative types (see Table 3), a strength of PLS-SEM [62]. Reflective constructs included MOB, lifetime adversity, social connectedness, resilience, subjective wellbeing, and subjective illbeing. Subjective illbeing was modelled as a higher-order reflective construct, comprising depression, anxiety, and stress as lower-order factors (see S2 File).

**Table 3:** Latent constructs and Loading factors for PLS-SEM.

Latent Variable	Type	Loading Factor (UK Biobank Measure/Questionnaire)
HRV	Formative	(a) 10s RMSSD (calculated from resting 10s ECG data)
Current Adversity	Formative	(a) Townsend deprivation index (ordinal variable ranging from -6.26 to 11.00; higher scores mean more deprivation, relating to the participant’s postcode at recruitment)
Meaning Oriented Behaviour	Reflective	(a) Belief that own life is meaningful (General wellbeing) (b) Belief that own life is meaningful (Happiness and subjective wellbeing) (c) Felt that life was not worth living (Self-harm behaviours; reverse scored) (d) Felt that life was not worth living (Harm behaviours; reverse scored)
Lifetime Adversity	Reflective	(a) Felt hated by family member as a child (Traumatic events) (b) Felt hated by family member as a child (Adverse life events) (c) Physically abused by family member as a child (Traumatic events) (d) Physically abused by family member as a child (Adverse life events) (e) Felt loved as a child (Traumatic events; reverse scored) (f) Felt loved as a child (Adverse life events; reverse scored)
Social Connectedness	Reflective	(a) Frequency of feeling in tune with people (Social situation) (b) Frequency of feeling isolated from others (Social situation; reverse scored) (c) Frequency of feeling left out (Social situation; reverse scored) (d) Frequency of feeling that lacks companionship (Social situation; reverse scored)
Resilience	Reflective	(a) Comes through difficult times with little trouble (Social situation) (b) Quick recovery from stressful events (Social situation) (c) Tendency to bounce back quickly after hard times (Social situation) (d) Hard to snap back when something bad happens (Social situation; reverse scored) (e) Tendency to take a long time to get over setbacks (Social situation; reverse scored)
Subjective Wellbeing	Reflective	(a) General happiness (Happiness and subjective wellbeing) (b) General happiness (General wellbeing) (c) Happiness (Mental health) (d) Happy over last week (Mood)
Subjective Illbeing	Higher Order/ Reflective	(a) Anxiety (b) Depression (c) Stress
Anxiety	Lower Order	(a) Feeling anxious, nervous, or on edge over the last 2 weeks (Health and wellbeing) (b) Not being able to control worrying over the last 2 weeks (Health and wellbeing) (c) Recent feelings of foreboding (Anxiety) (d) Recent feelings of foreboding (Anxiety and panic) (e) Recent feelings of nervousness or anxiety (Anxiety and panic) (f) Recent inability to stop or control worrying (Anxiety) (g) Recent inability to stop or control worrying (Anxiety and panic) (h) Recent worrying too much about different things (Anxiety and panic)
Depression	Lower Order	(a) Downhearted and depressed over last week (Mood) (b) Down in dumps over last week (Mood) (c) Feeling down, depressed, or hopeless over the last 2 weeks (Health and wellbeing) (d) Little interest or pleasure in doing things over the last 2 weeks (Health and wellbeing)
Stress	Lower Order	(a) Recent easy annoyance or irritability (Anxiety and panic) (b) Recent feelings of inadequacy (Depression) (c) Recent trouble relaxing (Anxiety) (d) Recent trouble relaxing (Anxiety and panic)

Reflective constructs assume that the latent variable causes variation in the observed indicators, which are expected to be interchangeable and highly correlated. In contrast, formative constructs are defined by their indicators, where each item captures a unique aspect of the construct and may not be correlated with other indicators. In this study, HRV (indexed via RMSSD) and current adversity (Townsend deprivation score) were modelled formatively, while all other latent variables were reflective.

Items were selected based on well-established theoretical models (e.g., the dual continua model, process-based therapy, and positive psychological frameworks) and confirmed through preliminary exploratory analyses. When the same question appeared in multiple UK Biobank questionnaires, each instance (labelled A or B) was kept as a separate indicator because it captured non-redundant variance. Construct reliability, convergent validity, and discriminant validity were assessed using standard criteria (see Table 2) [63–65]. Given our aim of exploring distinct pathways to wellbeing and illbeing, our measurement model includes indicators capturing both positive and negative facets of mental health, which naturally yield negative correlations. Since HTMT2 requires all indicator correlations to be positive, we opted for the traditional HTMT method, which utilises absolute values, to robustly assess discriminant validity under these conditions [63,66]. Each item’s factor loading was evaluated, with items loading above 0.70 retained, and those between 0.65 and 0.70 considered for retention if their removal negatively impacted internal consistency or convergent validity [63,67].

Formative latent variables included HRV and current adversity. HRV was measured using the root mean square of successive differences (RMSSD) from 10-second resting ECG recordings (see S3 File), while current adversity was indexed by Townsend deprivation scores, reflecting socioeconomic disadvantage based on participant postcodes.

### Network centrality analysis

To further explore the structural properties of our PLS-SEM, latent variable network was constructed based on the estimated path coefficients, yielding an adjacency matrix representing interrelations among key latent constructs. Three standard centrality indices were computed, degree, closeness, and betweenness, to quantify each construct’s local and global influence (see Table 2) [68]. These metrics are widely used in health psychology to identify influential hub variables. For example, degree centrality has helped pinpoint core symptoms in depression networks, that serve as effective intervention targets [69,70]. This network-based assessment of variable importance aligns with a biopsychosocial framework and recent work integrating psychological and physiological domains [71].

### Bayesian regression modelling

Bayesian regression was conducted using the pymc3 Python package [72] to estimate direct relationships between latent variable predictors (Lifetime Adversity, Resilience, Social Connectedness, MOB, HRV) and outcomes (Subjective Wellbeing and Illbeing), as identified in the PLS-SEM analysis. This approach allowed for modelling of the full posterior distributions for each parameter, capturing uncertainty and accommodating potential non-linear effects common in psychological data [73,74] (see Table 2).

Latent variable scores were scaled to a 0–1 range using min–max normalisation to ensure comparability across predictors [75]. The dataset was then randomly split into training (n = 4,024) and test (n = 4,023) subsets to support model validation and reduce risk of overfitting. We used non-informative priors to allow the large UK Biobank dataset to drive estimation of the posterior distributions [76]. Model estimation was performed using the No-U-Turn Sampler (NUTS), with two independent Markov Chain Monte Carlo (MCMC) chains, each running for 3,000 iterations (2,000 for adaptation and 1,000 for sampling), yielding 4,000 posterior samples overall. Convergence was assessed using the R-hat statistic and Effective Sample Size (ESS) thresholds (see Table 2) [77,78].

Bayesian regression modelling was conducted in two stages. First, six functional forms, linear, quadratic, cubic, exponential, logarithmic, and sigmoid, were tested for each predictor–outcome pair, from which the best-fitting form was selected using WAIC and LOO cross-validation scores [79]. In the second stage, the best-fitting functional relationships were incorporated into a single integrated Bayesian regression model to estimate the effects of all significant predictors simultaneously, while accounting for shared variance.

We assessed practical significance using the Region of Practical Equivalence (ROPE) [80]), treating parameters as meaningful if less than 5% of the posterior distribution fell within ±0.05 of zero. Model performance was assessed using the held-out test dataset, with predictive accuracy quantified via Bayesian R², Mean Absolute Error (MAE), and Root Mean Square Error (RMSE) [81].

## Results

### Partial-least-squares structural equation model

**Fig 1:**
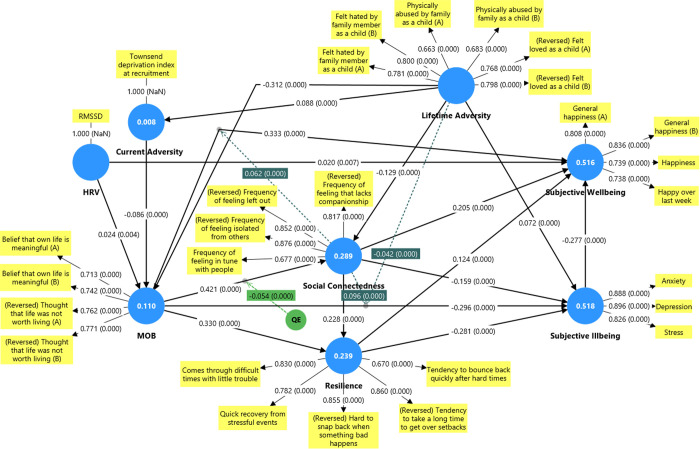
PLS-SEM (Full dataset).

PLS-SEM was applied to the full sample (N = 8,047; see Fig 1), which was evaluated through measurement and structural model assessments (see S4 File), then cross-validated via a random split-half procedure (see S5 File), confirming that all path estimates and explained variances were stable across subsamples. At the measurement level, the model satisfied every standard threshold: all indicator loadings ≥ 0.70 (items loading ≥ 0.65 were retained to preserve convergent validity [82]), composite reliabilities and Cronbach’s α exceeded 0.70, AVEs were above 0.50, VIFs remained below 5 to rule out multicollinearity [83], and all HTMT ratios fell under 0.85 [63]. Structurally, the model explained substantial variance, R² values were 0.11 for MOB, 0.29 for social connectedness, 0.24 for resilience, 0.52 for wellbeing, and 0.52 for illbeing, and exhibited predictive relevance (all Q² > 0 via PLS-Predict [84]; see S6 File). Global fit was acceptable (SRMR = 0.064 [85]; NFI = 0.78 [86]), consistent with PLS-SEM’s emphasis on explained variance (R²/Q²) over covariance-based fit indices [51,87]. Finally, all hypothesised paths achieved p < .001 under a two-tailed, 10,000-sample bootstrapping test [64,88].

### PLS-SEM Key findings

The PLS-SEM revealed significant direct, indirect, and total effects between our latent constructs (see S4 File for full path coefficients and effect summaries). HRV emerged as an upstream factor, with direct effects that enhanced MOB (β = 0.024, p < 0.005) and wellbeing (β = 0.020, p < 0.01), but not illbeing. However, HRV indirectly impacted on illbeing through MOB, significantly affecting both resilience (β = 0.010, p < 0.001) and social connectedness (β = 0.010, p < 0.001), subsequently reducing illbeing (β = -0.012, p < 0.001) and improving wellbeing (β = -0.015, p < 0.001).

MOB played a central role in this model, directly impacting on wellbeing (β = 0.333, p < 0.001) and illbeing (β = -0.296, p < 0.001). MOB facilitated a serial mediation pathway, enhancing social connectedness directly (β = 0.421, p < 0.001), and resilience directly (β = 0.330, p < 0.001) and indirectly (β = 0.096, p < 0.001) through social connectedness, which in turn contributed to wellbeing (β = 0.273, p < 0.001) and mitigates illbeing (β = -0.187, p < 0.001). Additionally, there was a notable quadratic effect of MOB on social connectedness (b = -0.054, p < 0.001), indicating non-linear influences, with implications for social integration and mental health.

Social connectedness and resilience both proved to be significant factors. Their total effects (combining direct and indirect influences) revealed that social connectedness positively impacted on wellbeing (β = 0.295, p < 0.001) and negatively impacted illbeing (β = -0.223, p < 0.001), with similar findings for resilience (wellbeing (β = 0.202, p < 0.001; illbeing, β = -0.281, p < 0.001). However, the interaction between social connectedness and MOB revealed unexpected complexities, while social connectedness generally amplified the positive effects of MOB on wellbeing (interaction effect β = 0.062, p < 0.001), it also revealed positive effects on and potential risks for illbeing (interaction effect β = 0.096, p < 0.001). This suggests that while social ties are generally beneficial, they may, under certain conditions (e.g., misalignment with personal values or overwhelming social expectations), contribute to psychological distress.

Lifetime adversity had a significant and pervasive impact, directly increasing illbeing (β = 0.072, p < 0.001) and current adversity (β = 0.088, p < 0.001), while negatively affecting MOB (β = -0.312, p < 0.001) and social connectedness (β = -0.129, p < 0.001). These reductions indirectly reduced resilience (β = -0.166, p < 0.001), resulting in total effects that reduced wellbeing (β = -0.252, p < 0.001) and increased illbeing (β = 0.255, p < 0.001). Lifetime adversity also moderated the effect of MOB on illbeing (interaction effect β = -0.042, p < 0.001), indicating that individuals with higher levels of adversity might experience a reduced protective effect of MOB against illbeing.

### Network centrality analysis

**Fig 2:**
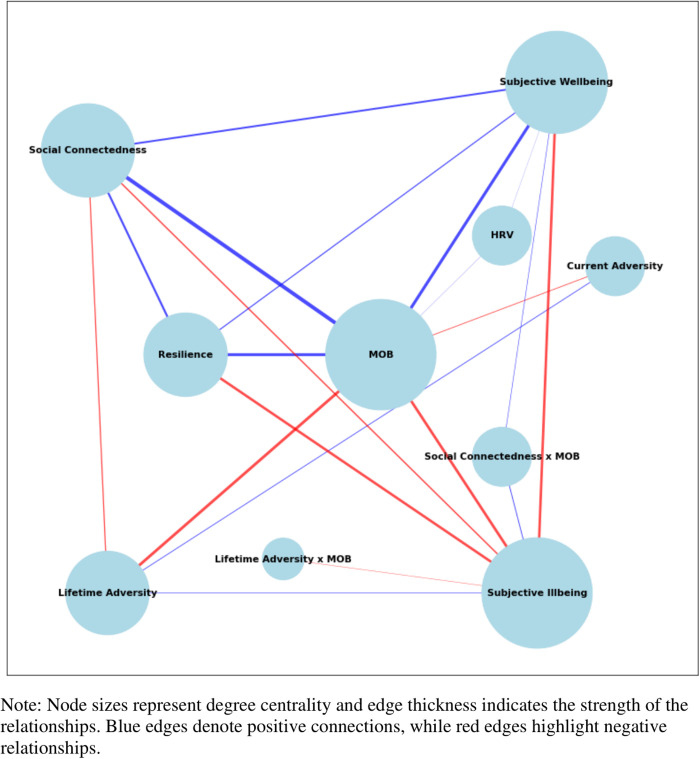
Network Centrality of Latent Variables in PLS-SEM.

The network centrality analysis (see Fig 2) revealed MOB as the most central construct, with the highest degree centrality (0.78) and notable closeness centrality (0.33). This underscores its pivotal role in influencing both subjective wellbeing and illbeing, and its high betweenness centrality (0.12) highlights its importance as a key mediator within the network.

HRV displayed moderate degree centrality (0.22), indicating, although less connected overall, that it remains an influential upstream factor due to its relative proximity to other key constructs within the network. Despite low betweenness centrality (<0.01), HRV’s role in the network is reflected in the PLS-SEM findings which shows that this variable initiates important pathways impacting on MOB and subsequently, other central variables including resilience and social connectedness.

Social connectedness and resilience had moderate degree centrality (0.56 and 0.44, respectively) and relatively high closeness centrality (0.62 and 0.58, respectively), indicating their roles as important supporting factors in the network (see S7 File for the full network centrality analysis results table).

### Bayesian regression modelling

To examine potential non-linear relationships between predictors and outcomes, Bayesian regressions were fitted to every significant direct path identified in the PLS-SEM. Six candidate functional forms were tested for each relationship, and the best-fitting form was selected based on model fit comparisons using WAIC and LOO cross-validation metrics (see S8 File; see Table 2). These relationships were then incorporated into an integrated model that generated posterior distributions for each parameter while adjusting for shared variance among predictors, thereby capturing their joint influence on wellbeing and illbeing within the overall PLS-SEM framework.

This approach quantified the strength and direction of effects and clarified their functional geometry, whether linear, quadratic, cubic, exponential, logarithmic, or sigmoid. Full parameter estimates, functional forms, and credibility intervals are reported below (see Table 4).

**Table 4:** Bayesian regression model estimates.

	Mean (*β*)	SD	hdi_3%	hdi_97%	ROPE (%)	ess_bulk	ess_tail	r_hat
Intercept for Subjective Wellbeing	0.499	0.018	0.464	0.531	N/A	1784	1414	1
Intercept for Subjective Illbeing	0.656	0.009	0.64	0.673	N/A	1754	1310	1
HRV (RMSSD) Impact on Wellbeing (Exponential)	0.01	0.008	-0.003	0.025	3.35	2117	1388	1
MOB Impact on Wellbeing (Cubic)	0.352	0.014	0.327	0.378	0	3361	1588	1
Social Connectedness Impact on Wellbeing (Cubic)	0.1	0.008	0.085	0.115	0	2599	1605	1
Resilience Impact on Wellbeing (Cubic)	0.074	0.013	0.05	0.099	0	2369	1510	1
Illbeing impact on Wellbeing (Logarithmic)	-0.499	0.021	-0.54	-0.462	0	2247	1433	
Lifetime Adversity Impact on Illbeing (Logarithmic)	0.12	0.014	0.093	0.147	0	2098	1409	1
MOB Impact on Illbeing (Cubic)	-0.147	0.015	-0.174	-0.117	0	2688	1428	1
Social Connectedness Impact on Illbeing (Cubic)	-0.149	0.008	-0.165	-0.135	0	2814	1506	1
Resilience Impact on Illbeing (Cubic)	-0.287	0.014	-0.311	-0.261	0	3276	1606	1
Variance (sigma) for Wellbeing	0.211	0.002	0.206	0.215	N/A	3626	1278	1
Variance (sigma) for Illbeing	0.235	0.003	0.229	0.24	N/A	3599	1517	1

Mean (β) represents the posterior mean estimate of the regression coefficient; SD indicates the standard deviation of the posterior distribution; hdi_3% and hdi_97% denote the lower and upper bounds of the highest density interval (HDI), respectively; ROPE (%) reflects the percentage of the posterior distribution within the region of practical equivalence; ess_bulk and ess_tail are the effective sample sizes for the bulk and tail of the posterior distribution, respectively; and r_hat is the Gelman-Rubin convergence diagnostic, with values close to 1 indicating satisfactory convergence.

MOB exhibited the strongest positive association with subjective wellbeing (β = 0.352, SD = 0.014, 95% HDI [0.327, 0.378], ROPE = 0%), with this relationship best modelled by a cubic function. This indicates complex, non-linear dynamics, potentially involving thresholds or reversals in effect strength at higher or lower levels of MOB.

Social connectedness (β = 0.100, SD = 0.008, HDI [0.085, 0.115], ROPE = 0%) and resilience (β = 0.074, SD = 0.013, HDI [0.050, 0.099], ROPE = 0%) also demonstrated significant positive effects on wellbeing, and both relationships were likewise best modelled by cubic functions. These results indicate that their benefits for wellbeing may be strongest at particular levels and may plateau or change beyond certain thresholds, supporting the need for nuanced and individualised intervention strategies.

The relationship between subjective illbeing and wellbeing was strongly negative (β = –0.499, SD = 0.021, HDI [–0.540, –0.462], ROPE = 0%) and was best described by a logarithmic function. This pattern indicates diminishing returns, as illbeing reduces, wellbeing improves most sharply at first, but the marginal gains taper off at lower levels of distress. This finding supports the dual continua model by showing that low illbeing does not equate to high wellbeing, thereby challenging traditional unipolar conceptions of mental health.

HRV showed a modest but meaningful exponential effect on wellbeing (β = 0.010, SD = 0.008, HDI [–0.003, 0.025], ROPE = 3.35%). While smaller than the psychosocial predictors, the exponential pattern suggests that wellbeing benefits increase disproportionately at higher levels of HRV, consistent with theoretical models of vagal functioning and self-regulation.

Lifetime adversity was positively associated with illbeing (β = 0.120, SD = 0.014, HDI [0.093, 0.147], ROPE = 0%), with the relationship best described by a logarithmic function. This indicates that initial increases in adversity have disproportionately large negative effects on mental health, while additional increases contribute progressively less, a threshold-type pattern often seen in trauma research.

MOB, social connectedness, and resilience also significantly predicted lower illbeing, each showing cubic relationships. MOB (β = –0.147, SD = 0.015, HDI [–0.174, –0.117], ROPE = 0%), social connectedness (β = –0.149, SD = 0.008, HDI [–0.165, –0.135], ROPE = 0%), and resilience (β = –0.287, SD = 0.014, HDI [–0.311, –0.261], ROPE = 0%) each contributed to reductions in distress, with their complex non-linear forms again suggesting that the effectiveness of each construct may vary depending on individual differences and current psychosocial context.

**Fig 3:**
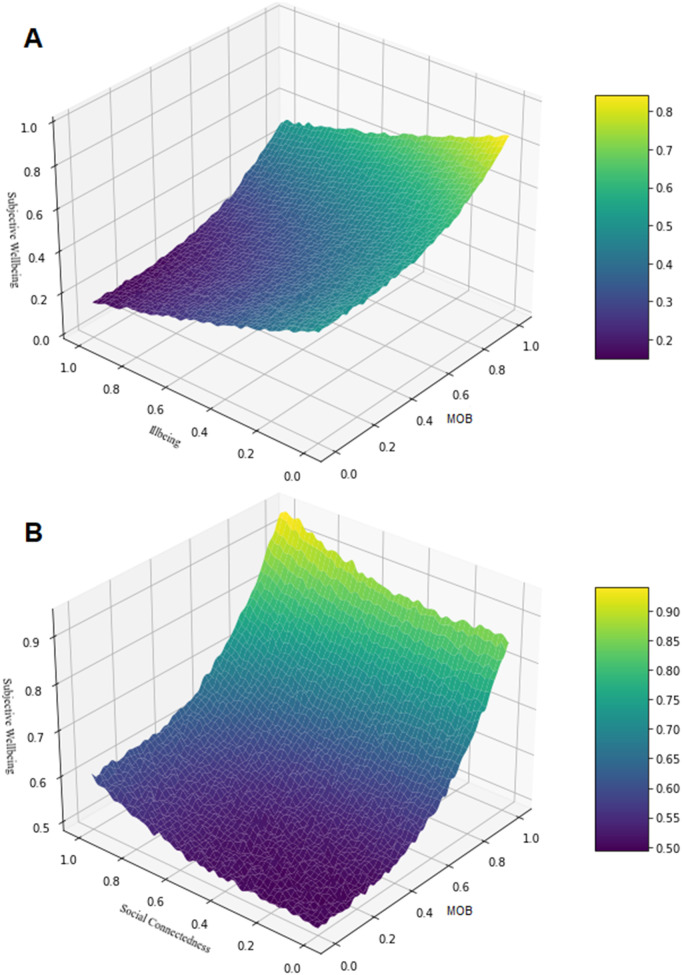
3D Surface Plots of: (a) Subjective Wellbeing predicted by MOB and Subjective Illbeing; (b) Subjective Wellbeing predicted by MOB and Social Connectedness.

The final integrated Bayesian regression model demonstrated strong convergence and reliability, with all R-hat values equalling 1.00 and effective sample sizes exceeding recommended thresholds. Applying the ROPE criterion, we found that all primary predictors had less than 5% of their posterior distributions within the ROPE interval around zero, indicating effects that were both statistically and practically meaningful. Key non-linear interactions are visualised in bivariate surface plots (see Fig 3).

To assess predictive performance, we evaluated the model using a held-out test dataset (n = 4,023). The Bayesian R² was 0.404 for subjective wellbeing and 0.543 for illbeing, indicating moderate to strong predictive power. Prediction error was low, with mean absolute error (MAE) values of 0.162 (wellbeing) and 0.179 (illbeing), and root mean square error (RMSE) values of 0.203 and 0.220, respectively. These results confirm that the model not only achieved robust estimation but also generalised well to unseen data. Additional diagnostics and WAIC/LOO model selection procedures are reported in S8–S9 Files.

### Summary of key findings

**RQ1** Subjective wellbeing and illbeing are related but distinct constructs, with a logarithmic inverse relationship observed between them. Predictors demonstrated distinct patterns of association with each outcome. Meaning-oriented behaviour (MOB), resilience, and social connectedness predicted both wellbeing and illbeing through non-linear cubic functions. Lifetime adversity predicted illbeing only, via a logarithmic function, while HRV predicted wellbeing only, with an exponential function. These patterns were confirmed in both structural and Bayesian regression models.

**RQ2** MOB and HRV emerged as distinct but complementary influences on mental health. MOB showed strong direct associations with both increased wellbeing and reduced illbeing, with non-linear effects observed across its full range. HRV, while a weaker predictor, showed a non-linear exponential relationship with wellbeing but did not significantly predict illbeing directly. However, SEM analyses indicated that HRV influenced illbeing indirectly via its effects on MOB, resilience, and social connectedness. These results highlight complementary behavioural and physiological pathways to wellbeing, with HRV exerting its influence indirectly through psychosocial mechanisms.

**RQ3** Evidence of mediation and moderation was observed across multiple paths. Social connectedness, resilience, and adversity all contributed to indirect effects between core predictors and outcomes. For example, the relationship between MOB and illbeing was moderated by social connectedness, and resilience partially mediated the effects of adversity. These complex interrelations were captured in the network analysis and supported by path coefficients from the PLS-SEM.

## Discussion

This study explored the pathways and relationships between various predictors of subjective wellbeing and illbeing, using a multipronged analytical approach that included PLS-SEM, Network Centrality Analysis, and Bayesian Regression Modelling. These methods allowed us to identify and clarify the direct, indirect, and non-linear pathways through which biopsychosocial factors influence mental health outcomes.

### Distinguishing wellbeing and illbeing and functional relationships with key predictors

First, we address whether subjective wellbeing and illbeing are distinct constructs, and the functional nature of their relationship, including how predictors (HRV, MOB, resilience, social connectedness, adversity) relate to these outcomes. This distinction was supported through PLS-SEM, which demonstrated that wellbeing and illbeing are best represented as separate latent constructs with overlapping but non-identical predictor patterns. Bayesian regression modelling further showed that the relationship between these outcomes followed a logarithmic form, whereby initial reductions in illbeing led to substantial improvements in wellbeing, while such gains tapered off as illbeing decreased further, underscoring the complex non-linear interplay between the two constructs. Combined with evidence that wellbeing and illbeing arise from partially distinct pathways, this suggests that interventions focused solely on symptom reduction may be insufficient for addressing all aspects of wellbeing. Complementary approaches that explicitly target the enhancement of wellbeing (e.g., positive psychotherapy [10,21]) alongside those aimed at reducing illbeing (e.g., cognitive behavioural therapy), may therefore be required.

Differences in predictor effects across the PLS-SEM and Bayesian models further illustrate that the pathways to wellbeing and illbeing are not simply symmetrical, but reflect distinct and context-sensitive dynamics. While both models showed that MOB, social connectedness, and resilience positively influence wellbeing and negatively impact illbeing, the non-linear patterns observed, particularly the cubic trajectories for MOB and social connectedness, indicate that their effects vary across different levels of wellbeing and illbeing. This highlights the importance of considering such non-linear dynamics when designing interventions, as these findings suggest that intervention effectiveness may vary according to an individual’s baseline mental health status, reinforcing a need for adaptive, context-sensitive approaches tailored to different levels of wellbeing or illbeing.

Together, these results extend support for the dual continua model of mental health, which conceptualises wellbeing and illbeing as distinct but interconnected [37,89,90]. By identifying divergent predictor-outcome pathways and distinct functional forms, such as the selective effects of HRV and adversity, and the logarithmic relationship between wellbeing and illbeing, we provide evidence that these constructs warrant separate, yet complementary, intervention strategies [5]. This has important implications for clinical and rehabilitation contexts, including chronic conditions, such that efforts to enhance wellbeing are complementary and have additional benefits over and above reducing distress, as has been shown in recent research with those living with acquired brain injury [24,26]. The non-linear effects observed for MOB and social connectedness reinforce the need for flexible, context-sensitive approaches that are responsive to an individual’s mental health and psychosocial functioning.

### Upstream influences: The roles of meaning-oriented behaviour and vagal function

A core aim of this study was to examine how HRV and MOB contribute to mental health outcomes. MOB demonstrated the strongest total effects on both wellbeing and illbeing in the PLS-SEM, and it emerged as the most central node in the network analysis, underscoring its integrative role across the broader psychosocial system. It exerted both direct effects on mental health and indirect effects via social connectedness and resilience. HRV also played a key upstream role, showing positive associations with wellbeing, and indirect links with lower illbeing through its positive influence on MOB. These findings suggest that while HRV may reflect the physiological capacity for regulation, MOB appears to channel this capacity into value-based behavioural engagement, which is associated with downstream psychosocial processes.

Bayesian modelling revealed that HRV’s association with wellbeing followed an exponential trajectory, with psychological benefits increasing more rapidly at higher levels of HRV. While this non-linear pattern does not provide evidence for a particular threshold, it is consistent with theoretical models suggesting that observable vagal influences on wellbeing may only emerge beyond a certain physiological level [14,91]. Longitudinal evidence indicates that individuals with higher baseline HRV derive greater emotional benefits from affective interventions, suggesting that vagal tone may facilitate the emergence of an upward spiral in wellbeing once a sufficient level is reached [92]. In this view, increased HRV may initiate a positive feedback loop, whereby enhanced vagal function promotes psychosocial resources such as social connectedness and resilience, which in turn support behaviours that sustain vagal tone and psychological wellbeing [93]. This interpretation is further supported by evidence showing reciprocal influences between positive emotion, social connection, and vagal tone [92,94]. Although the cross-sectional design limits causal inference, these models offer plausible mechanisms for the exponential association observed. Future research could test for true threshold effects using longitudinal models that allow for changes in the slope of association at specific points (e.g., segmented or spline-based mixed-effects models), and examine causality through interventions that target vagal tone (e.g., HRV biofeedback). Such studies would help clarify whether specific HRV levels are necessary to initiate wellbeing improvements, and whether dynamic, self-reinforcing processes underlie this non-linear pattern.

MOB also showed a non-linear association with both wellbeing and illbeing, with Bayesian modelling identifying a cubic trajectory, indicating that its observed association with mental health outcomes varied in strength across the range of MOB. In the PLS-SEM analysis, MOB was positively associated with social connectedness, and this relationship also included a significant quadratic component, forming an inverted U-shaped curve. This suggests that while greater MOB generally fosters stronger social ties, the strength of this association peaks at moderate levels and is attenuated at both lower and higher levels. One interpretation is that social connectedness may be maximised when perceived social expectations are broadly coherent with one’s personal values. At low levels of MOB, such alignment may be lacking, while at very high levels, rigid value pursuit may lead to interpersonal friction. This aligns with evidence that inflexible or overcontrolled goal pursuit can undermine wellbeing and strain social relationships, particularly when values conflict with social norms or expectations [95,96]. In this context, MOB may serve not only as a key determinant of mental health, but also as a mechanism through which physiological regulation and psychosocial functioning interact to support wellbeing.

Together, these findings highlight HRV and MOB as key correlates of mental health outcomes, operating through social connectedness and resilience. HRV may serve as a physiological foundation, while MOB facilitates behavioural alignment with meaning and values. These results support interventions that target both vagal regulation and value-driven behaviour to optimise wellbeing [14,97]. The possibility of mutually reinforcing effects between HRV and psychosocial processes represents an important direction for future longitudinal and experimental research [92,94].

### Contextual pathways: Mediation and moderation by adversity, resilience, and social connectedness

Finally, we examine the mediating and moderating effects of adversity, social connectedness, and resilience on these PLS-SEM pathways. Lifetime adversity had a significant direct effect on subjective illbeing but not wellbeing. It also directly affected MOB, social connectedness, and current adversity, leading to substantial total indirect effects that subsequently reduced wellbeing. Although lifetime adversity did not directly impact resilience, it influenced pathways to wellbeing and illbeing through its negative effects on MOB and social connectedness, which in turn affected resilience. Similarly, current adversity negatively affected MOB, indirectly reducing social connectedness and resilience, underscoring the immediate impact of socio-economic stressors on the sense of meaning and purpose. Ultimately, these results highlight the centrality of MOB in buffering against the negative impacts of adversity, emphasising their critical role in maintaining mental health through their influence on social connectedness and resilience.

In our analysis, social connectedness and resilience emerged as key downstream mediators. Specifically, social connectedness not only mediated the relationship between MOB and mental health outcomes but also served as a precursor to resilience, which further transmitted these effects. Bayesian regression modelling revealed that both social connectedness and resilience exhibit significant cubic effects on wellbeing and illbeing, with resilience showing a particularly strong negative impact on illbeing.

Three significant moderating effects were observed in the PLS-SEM. The interaction between lifetime adversity and MOB was significant for illbeing but not for wellbeing, suggesting that while MOB can help mitigate the adverse effects of past trauma on mental distress, it does not necessarily enhance positive aspects of mental health in the same context. The moderating effects of social connectedness were also significant, enhancing the positive impact of MOB on wellbeing, potentially reflecting improvements in life satisfaction and happiness when one’s social ties align with their core values. However, the same interaction was associated with increased illbeing, indicating that high levels of social connectedness may, under certain conditions, counteract the benefits of MOB, possibly due to stress or conflict arising from misaligned social expectations. Overall, these findings indicate that the effects of MOB and social connectedness on mental health are contingent on both their interaction and broader contextual factors. While MOB generally enhances wellbeing and reduces illbeing, the amplification provided by strong social ties can sometimes counteract these benefits. This pattern aligns with literature suggesting that although social support is typically beneficial, it can also become a source of stress when it conflicts with personal values or autonomy [98,99].

These insights underscore the need for tailored mental health interventions that foster meaningful, value-congruent social connections, particularly for individuals who may find social interactions stressful, such as those with depression who tend to favour isolation or introverted individuals who value independence [100,101]. By taking such individual differences into account, interventions can more effectively reduce illbeing and promote wellbeing, ensuring that social connections serve as a supportive, rather than undermining, factor.

## Implications

The findings of this research have practical implications for clinicians and public health professionals, pointing to the importance of recognising non-linear associations between wellbeing and illbeing. These patterns are consistent with frameworks that emphasise personal meaning in a biopsychosocial context, such as the Power Threat Meaning Framework [102] and extended evolutionary meta-model [103]. PLS-SEM revealed non-linear associations between MOB, social connectedness, resilience, and mental health outcomes. While not an intervention study, these findings suggest that strategies tailored to individuals’ values, psychosocial resources, and life contexts may be more effective than uniform approaches [104–106].

HRV emerged as an upstream physiological marker of wellbeing, aligning with the GENIAL meta-framework that links vagal function with self-regulation, social engagement and connection with nature [3,13]. Strategies that raise vagal tone, including balanced diet, progressive physical activity and stress-management techniques [107,108], provide an opportunity to enhance the alignment between physiological capacity and valued action, thereby reinforcing resilience and social connection [12,109].

In this study, MOB was both the most central node and the strongest predictor of higher wellbeing and lower illbeing. These results resonate with third-wave therapies such as ACT and positive psychotherapy, which foster psychological flexibility (a construct that is conceptually and empirically associated with greater wellbeing in ACT models) through values-based action [12,110,111].

The observed non-linear trends suggest that wellbeing gains are greatest when factors such as social connection, purposeful activity, and challenge are within an optimal range. This is consistent with evidence that face-to-face interaction enhances wellbeing in older adults up to a point [112], that volunteering is beneficial when moderately demanding [113], and that resilience is highest among those who have experienced moderate, rather than minimal or extreme, adversity [114]. Public health efforts should therefore aim to support balanced engagement, rather than assuming that more is always better.

## Limitations

Several limitations warrant mention. HRV was calculated from the 10-second resting ECG traces from the UK Biobank, so the measure captures only a brief snapshot of autonomic activity. However, prior work indicates that ten-second RMSSD correlates well with longer recordings [115], and our own supplementary analysis supports these findings (R² = 0.507, mean bias < 20 ms for 80% of participants; S3 File). Furthermore, all structural paths involving HRV replicated in our random split-half SEM analysis, suggesting that any residual measurement noise was insufficient to meaningfully alter the pattern of associations.

Missing data constituted another limitation in this study. On average, 64% of values were missing across the 37 questionnaire items and ECG data, and because these gaps occurred across different participants, only 8,047 individuals had complete data and were included in the final analyses. Consistent with previous UK Biobank SEM research [52,58], we adopted a complete-case analysis approach to ensure coherent estimation of latent constructs and structural paths within a unified model. This strategy allowed comparability across all three analytic models but resulted in a cohort that was older (mean age = 64.8 ± 7.7 years) and predominantly White British. While this reflects the wider demographic profile of a rapidly growing proportion of the UK’s population (adults aged 65 + are projected to approach one quarter of residents by 2050 [116]), it introduces limitations to generalisability, particularly as completeness was likely non-random and may have constrained the distributional range of key psychological constructs [117].

To evaluate the internal consistency of our constructs and whether our model generalises across key demographic strata within UK Biobank, we conducted measurement invariance testing using the Measurement Invariance of Composites (MICOM) procedure, followed permutation multigroup comparisons (see S10 File) [118]. MICOM confirmed compositional invariance for all six latent variables across age (youngest vs oldest quartiles: 40–50 vs 62–70 years), sex (female vs male), and ethnicity (White British vs combined UK Ethnic Minority participants), indicating that constructs were measured equivalently across groups. The multigroup analysis showed that while all structural paths remained statistically significant and directionally consistent across groups, a subset of paths differed in strength (6 by age, 2 by sex, and 5 by ethnicity). This indicates that while the model structure is broadly stable, some associations vary by demographic context. These findings support generalisability within UK Biobank, but further research will be required to determine external validity in younger, more diverse, or non-UK populations.

Finally, this study is cross-sectional and based on secondary data, so causal statements must remain tentative. Reflecting on this issue in more detail, we considered plausibility with the Bradford Hill criteria (Table 5) [119,120]. Future longitudinal and intensive within-person studies [121] are now required to establish temporal ordering and confirm dynamic non-linearities.

**Table 5:** Reflection on results of our study through the lens of the Bradford Hill Criteria.

Bradford Hill Criterion	Evidence from the study
Strength of Association	The PLS-SEM model explained a substantial proportion of variance in both wellbeing (R² = 0.518) and illbeing (R² = 0.516), indicating strong explanatory power. Standardised path coefficients for key predictors were consistently moderate to large in magnitude (e.g., β > 0.30 for MOB), and all were statistically significant (p < .001). Bayesian regression analyses corroborated these findings, with all key predictors showing high posterior certainty and practical significance (ROPE = 0% for all psychosocial variables). Together, these results support the interpretation that key psychosocial predictors meaningfully influence wellbeing and illbeing outcomes, consistent with our theoretical model. However, these associations remain observational and may reflect residual confounding or bidirectional effects.
Consistency	The study’s findings were consistent across multiple validation methods. Findings from PLS-SEM analysis on full sample (n = 8,047) was confirmed in a random split-half procedure, yielding nearly identical path coefficients and R² values in both randomly selected subsamples. Bootstrapping with 10,000 resamples produced narrow confidence intervals, and PLS-Predict analyses showed that all endogenous constructs had Q² values above zero. The Bayesian regression further reinforced these results; model selection was guided by cross-validation metrics (WAIC and LOO), and convergence diagnostics (R-hat ≈ 1, high effective sample sizes) confirmed the reliability of the posterior estimates. Finally, predictive performance on a held-out test dataset, evidenced by low MAE and RMSE values, demonstrated that the model generalises well to new data.
Specificity	Each predictor showed specific patterns of influence on wellbeing vs illbeing. HRV was associated with increased wellbeing (both directly and indirectly via MOB, resilience, and social connectedness), but showed no direct association with illbeing, which was primarily related to lifetime adversity (direct β = 0.072, p < 0.001) and its adverse effect on MOB and social connectedness. This separation suggests distinct influence pathways rather than a single common mechanism. Discriminant-validity tests (HTMT ≤ 0.75) confirmed that wellbeing and illbeing are statistically separable constructs. This supports construct-level specificity within the model.
Temporality	While the current study is cross-sectional, the theoretical model imposes a logical temporal order, guided by existing literature. Antecedent factors (e.g., lifetime adversity and HRV) are proposed to influence mediators (MOB, resilience, social connectedness), which are, in turn, proposed to shape wellbeing and illbeing outcomes. While this ordering aligns with previous longitudinal studies, the absence of time-separated measurements in the current data precludes empirical confirmation of sequence. As such, temporal precedence cannot be established, meaning a potential for reverse or reciprocal causality.
Biological Gradient (Dose–Response)	Bayesian regression analyses revealed non-linear dose–response relationships. HRV’s association with wellbeing was best described by an exponential function, whereby benefits increased disproportionately at higher levels of vagal tone. In contrast, the inverse association between illbeing and wellbeing followed a logarithmic form, with initial reductions in illbeing yielding substantial improvements in wellbeing that plateaued at lower levels of distress. In addition, psychosocial predictors such as MOB, resilience, and social connectedness exhibited cubic dynamics, and a significant quadratic effect was observed for MOB on social connectedness, highlighting graded and complex dose–response effects. These patterns are observational and cannot confirm underlying mechanisms without temporal or experimental data.
Plausibility	The proposed pathways are well grounded in both biological and psychological theory. HRV is an established marker of vagal tone and stress regulation, supporting emotional stability and social engagement. MOB is grounded in eudaimonic wellbeing theory and underpins modern therapeutic approaches like ACT, which emphasise value-driven behaviour, psychological flexibility, and meaning-making. These mechanisms also align with biopsychosocial frameworks (e.g., the GENIAL model), suggesting that it is plausible that higher HRV and stronger meaning-oriented behaviour enhance wellbeing-related outcomes.
Coherence	The results are internally consistent and congruent with the broader literature. Positive factors (HRV, MOB, resilience, social connectedness) correlate with improved wellbeing, while adverse factors (current/lifetime adversity) are linked to increased illbeing. Nuanced findings, such as the quadratic effect of MOB on social connectedness and the paradoxical moderation by social connectedness on the relationship between MOB and illbeing, are consistent with previous work suggesting that social connectedness, while generally beneficial, may under certain conditions undermine wellbeing. For example, when social roles or expectations conflict with personal values or an individual’s preference for autonomy. These effects align with established psychological models that emphasise the contextual and relational nature of mental health.
Experiment	No experimental manipulation was performed and attributions relating to causality must therefore be tempered. However, the study employed robust observational methods including PLS-SEM (with bootstrapping and split-half validation) and Bayesian regression (with cross-validation and predictive performance analysis), which provide a strong basis for formulating testable hypotheses for future experimental or longitudinal research.
Analogy	The observed relationships mirror findings from related research. For example, the disproportionate benefit of higher HRV on wellbeing in our study aligns with psychophysiological evidence showing that even modest increases in vagal tone can produce meaningful improvements in mental health [14]. Similarly, the buffering effects of MOB, resilience, and social connectedness are comparable to protective factors identified in stress and trauma research, supporting the generalisability of these mechanisms [23,24]. While these analogies increase conceptual support, they do not constitute direct evidence of causality.

## Conclusion

Overall, the combined insights from PLS-SEM and Bayesian regression modelling provide a novel and comprehensive understanding of the multifaceted nature of wellbeing and illbeing. Analyses revealed complex, non-linear dynamics among key constructs, highlighting the critical roles of MOB, resilience, and social connectedness in enhancing wellbeing and mitigating illbeing. Our findings also demonstrate that HRV, as an upstream psychophysiological marker, directly influences MOB and indirectly affects social connectedness and resilience, thereby reducing illbeing. These results underscore the necessity for sophisticated modelling approaches to capture the inherent complexity of mental health pathways. Further research is needed to validate and extend these findings in younger and more diverse samples, and using complementary methods, such as covariance-based SEM and longitudinal designs. This research agenda lays a strong foundation for enhancing wellbeing and reducing illbeing across diverse populations, providing a robust framework for future personalised mental health interventions and policies.

## Supporting information

S1 Checklist STROBE checklist. (S1_Checklist.PDF)

S1 File Conceptual model diagram used to guide latent construct development. (S1_File.PDF)

S2 File Description of the subjective illbeing latent factor and its item structure. (S2_File.PDF)

S3 File Details of ECG processing and HRV (RMSSD) calculation from 10s recordings. (S3_File.PDF)

S4 File Full PLS-SEM path coefficients and effect size tables. (S4_File.PDF)

S5 File Split-half validation results and model robustness checks. (S5_File.PDF)

S6 File Predictive relevance statistics (Q²) for key constructs from PLSPredict. (S6_File.PDF)

S7 File Centrality metrics from network analysis (degree, closeness, betweenness). (S7_File.PDF)

S8 File Model selection criteria (WAIC and LOO) for functional form comparisons. (S8_File.PDF)

S9 File Bayesian regression diagnostics, surface plots, and ROPE analyses. (S9_File.PDF)

S10 File Permutation Multigroup Analysis. (S10_File.PDF)
