# Supplementary material for: Pathways, predictors and paradoxes of illbeing and wellbeing in older adults: Insights from a UK Biobank study
Source: PLOS Ment Health. 2025 Sep 3;2(9):e0000336. doi: 10.1371/journal.pmen.0000336 (PMC12798268; doi:10.1371/journal.pmen.0000336)
Supplement: S1 File — (S1_File.PDF) [file pmen.0000336.s002.pdf]

Supplementary 1 - Conceptual model

Figure 1:
Conceptual PLS-SEM

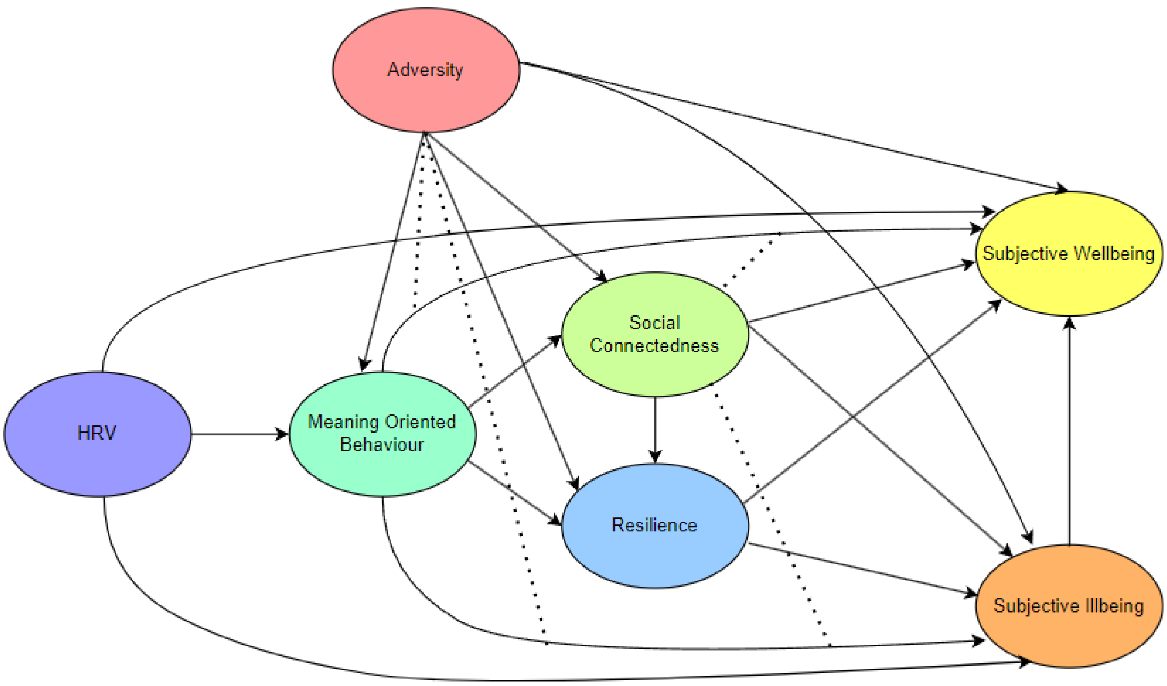

Note: Solid lines represent predicted direct relationships based on the literature review, and dotted lines represent predicted moderating effects.
