# Supplementary material for: Pathways, predictors and paradoxes of illbeing and wellbeing in older adults: Insights from a UK Biobank study
Source: PLOS Ment Health. 2025 Sep 3;2(9):e0000336. doi: 10.1371/journal.pmen.0000336 (PMC12798268; doi:10.1371/journal.pmen.0000336)
Supplement: S2 File — (S2_File.PDF) [file pmen.0000336.s003.pdf]

Supplementary 2 - Higher-Order Latent Variable for Subjective Illbeing

When constructing the latent variable for subjective illbeing, we identified a significantly larger number of conceptually relevant questionnaire items related to illbeing than any other construct in the model. However, when these items were loaded onto a single latent variable, the model exhibited poor composite reliability (CR < 0.7) and low factor loadings (indicator loadings < 0.7), indicating issues with construct validity. To resolve this issue and retain as much useful data as possible, we constructed a higher-order construct for subjective illbeing, for which the items were used to create three first-order constructs: anxiety, depression, and stress. The higher-order latent variable was specified using a reflective-formative type model, where the first-order latent variables (anxiety, depression, and stress) are reflective indicators of the higher-order construct [1]. This approach is appropriate given that subjective illbeing is understood to be manifested in part through these distinct yet related dimensions [2].

References

1. Becker J-M, Klein K, Wetzels M. Hierarchical latent variable models in PLS-SEM: guidelines for using reflective-formative type models. Long range planning. 2012;45(5-6):359-94.

2. Salari N, Hosseinian-Far A, Jalali R, Vaisi-Raygani A, Rasoulpoor S, Mohammadi M, et al. Prevalence of stress, anxiety, depression among the general population during the COVID-19 pandemic: a systematic review and meta-analysis. Globalization and health. 2020;16:1-11.
