# Supplementary material for: Pathways, predictors and paradoxes of illbeing and wellbeing in older adults: Insights from a UK Biobank study
Source: PLOS Ment Health. 2025 Sep 3;2(9):e0000336. doi: 10.1371/journal.pmen.0000336 (PMC12798268; doi:10.1371/journal.pmen.0000336)
Supplement: S3 File — (S3_File.PDF) [file pmen.0000336.s004.pdf]

Supplementary 3 - ECG heart-rate variability extraction

Biobank bulk datasets (including raw ECG data) are accessible via the Biobank Research Analysis Platform, which allows for online data analysis using Python with Jupyter Labs notebooks on their servers. Resting 12-lead ECGs (using Cardiosoft v6 program, GE Healthcare, Chicago, USA) were available for 67,311 Biobank participants. The duration of all signals in the dataset was 10 seconds, with a sampling frequency of 500 Hz, and a recording resolution of 5uv. We pre-processed and analysed the raw ECG signals from the 8,047 participants included in our structural equation model, to derive HRV (RMSSD) and mean resting heart rate.

The ECG waveform data was extracted from the Biobank dataset, which is stored within their bulk repository as .xml files. The 12-lead ECG data was extracted using the xml.etree Python package and converted into an array for further analysis.

We then applied a Butterworth band-pass filter between 0.5 and 50 Hz, to remove baseline wander artifacts and high-frequency noise. Next, we applied an r-peak detection method from the NeuroKit2 Python toolbox for neurophysiological signal processing [1, 2] as well as an adaptive threshold amplitude filter set to 0.25%. The code was also modified to utilise the NeuroKit2 artifact correction method in cases where RMSSD was found to exceed 250ms.

Internal validation

To test the validity of our analysis, and the 10-second RMSSD measurement of heart rate variability (10sHRV) as a predictor of a subject's broader HRV, we compared the results of calculating RMSSD from 10-second and 5-minute slices of PhysioNets publicly available ECG data [3]. We used the Autonomic Aging ECG dataset [4], which has a sampling frequency of 1000 Hz, and a recording resolution of 16uv.

Initially, Python code was employed to slice the data into the first 10 seconds and 5 minutes of each recording. Subsequent data preprocessing steps included the removal of participants with missing data, exclusion of participants with mean heart rates (HR) outside the range of 60-100 beats per minute, and the filtering out of RMSSD values greater than 150 to ensure data quality. Our method was then applied to detect RR peaks and calculate RMSSD for each participant and recording length.

A linear regression analysis was conducted to assess the association between the calculated 10sHRV and 5-minute HRV. The analysis revealed a significant positive association, with a beta coefficient of 0.707 ($p < 0.00001$) and an R-squared value of 0.507. This indicates that 50.7% of the variance in the 5-minute HRV is explained by the 10-second measurement, suggesting that the 10-second RMSSD measurement is a reliable predictor of an individual's broader HRV.

We observed a bias of -3.89, indicating a slight underestimation of HRV using the 10-second RMSSD method. On average, the RMSSD values from the 10-second segments were 3.89 units lower than those from the 5-minute segments. To understand the practical significance of this difference, the absolute mean difference for 80% of participants was calculated and found to be 19.71 units. This suggests that for 80% of the participants, the absolute difference between the 10sHRV and 5-minute HRV measurements was less than 19.71 units, supporting the reliability of the 10-second RMSSD as a practical alternative for HRV estimation when longer recordings are not feasible.

Figure 2:
Bland-Altman Plot illustrating the agreement between short (10s) and long (5-minute) RMSSD measurements

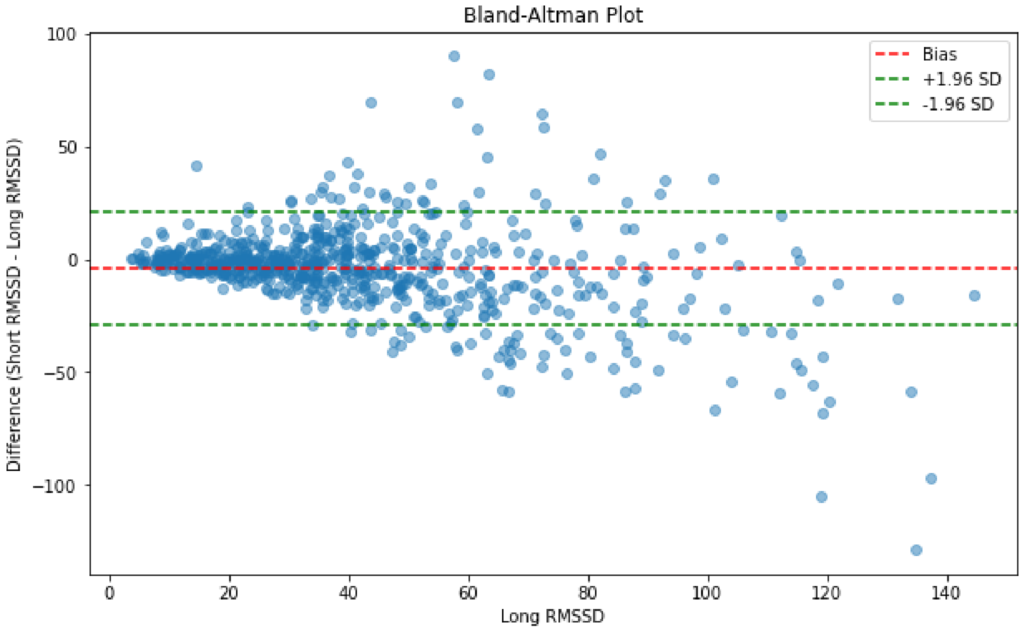

External validation

To further validate the reliability of the 10-second RMSSD measurement, we compared the results of our original method with an alternative approach using PhysioNet's Cardiovascular Signal Toolbox. The same dataset from PhysioNet's publicly available ECG data was used for consistency. The alternative method involved pre-processing and analysing the ECG data using PhysioNet's Cardiovascular Signal Toolbox, which provided a standardised way to calculate RMSSD.

Descriptive statistics for this method indicated that for the 10-second segments, the mean RMSSD was 39.00 (SD = 26.24), and for the 5-minute segments, the mean RMSSD was 44.42 (SD = 27.05). A linear regression analysis for the alternative method showed a beta coefficient of 0.634 ($p < 0.00001$) and an R-squared value of 0.378, indicating a significant but weaker association compared to the original method. The bias was -5.42, and the absolute mean difference for 80% of participants was 22.83 units.

Figure 3:
Bland-Altman Plot illustrating the agreement between short (10s) and long (5-minute) RMSSD measurements

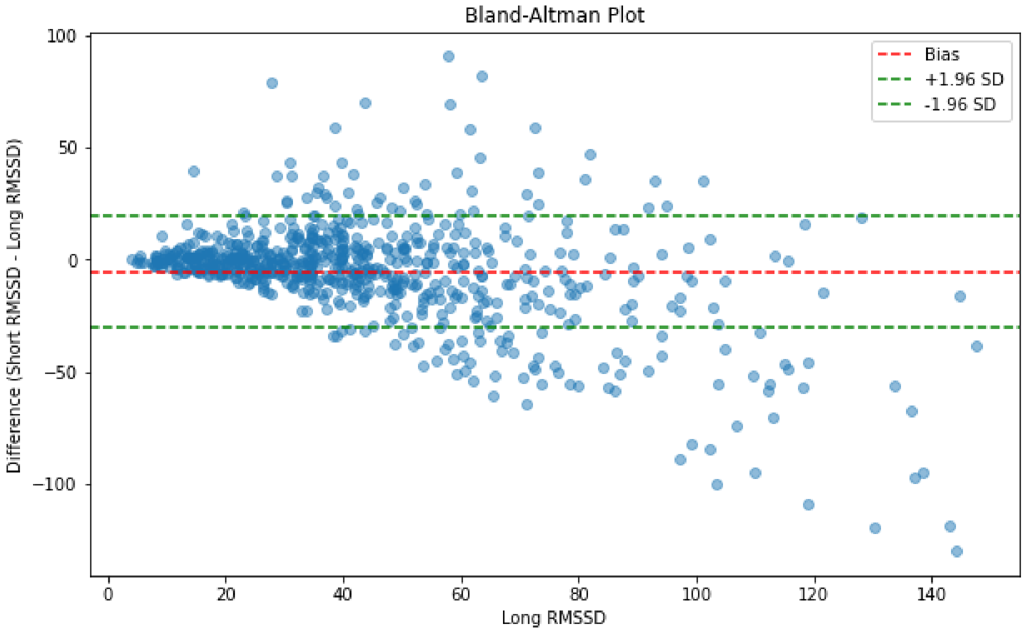

Both methods demonstrated a significant positive association between the 10-second and 5-minute RMSSD measurements, confirming the potential of short-term measurements to predict longer-term HRV. However, several key differences were noted. The original method showed a stronger association, with an R-squared value of 0.507 compared to 0.378 for the alternative method. This suggests that the original method explains a greater proportion of the variance in the 5-minute HRV. The bias was slightly lower in the original method (-3.89) compared to the alternative method (-5.42), indicating less systematic underestimation of HRV. The original method had a smaller absolute mean difference for 80% of participants (19.71 units) compared to the alternative method (22.83 units), indicating more consistency and closer agreement between short-term and long-term measurements.

References

1. Makowski D, Pham T, Lau ZJ, Brammer JC, Lespinasse F, Pham H, et al. NeuroKit2: A Python toolbox for neurophysiological signal processing. Behav Res Methods. 2021;53(4):1689-96. Epub 20210202. doi: 10.3758/s13428-020-01516-y. PubMed PMID: 33528817.
2. Brammer JC. Biopeaks: A graphical user interface for feature extraction from heart- and breathing biosignals. Journal of Open Source Software. 2020;5(54):2621.

3. Goldberger AL, Amaral LA, Glass L, Hausdorff JM, Ivanov PC, Mark RG, et al. PhysioBank, PhysioToolkit, and PhysioNet: components of a new research resource for complex physiologic signals. Circulation. 2000;101(23):E215-20. doi: 10.1161/01.cir.101.23.e215. PubMed PMID: 10851218.
4. Schumann A, Bar KJ. Autonomic aging - A dataset to quantify changes of cardiovascular autonomic function during healthy aging. Sci Data. 2022;9(1):95. Epub 20220323. doi: 10.1038/s41597-022-01202-y. PubMed PMID: 35322044; PubMed Central PMCID: PMCPMC8943176.
