# Supplementary material for: Pathways, predictors and paradoxes of illbeing and wellbeing in older adults: Insights from a UK Biobank study
Source: PLOS Ment Health. 2025 Sep 3;2(9):e0000336. doi: 10.1371/journal.pmen.0000336 (PMC12798268; doi:10.1371/journal.pmen.0000336)
Supplement: S4 File — (S4_File.PDF) [file pmen.0000336.s005.pdf]

Supplementary 4 - PLS-SEM Results

Measurement model assessment

Indicator loadings were assessed, with most surpassing the 0.7 thresholds, items with loadings between 0.65 and 0.7 were retained to preserve internal consistency and convergent validity [1], variance inflation factor (VIF) assessments demonstrated no multicollinearity issues, with all values well below 5 [2]. Internal consistency (Cronbach's Alpha) and Composite Reliability (Rho-A; Rho-C) were confirmed, as each endogenous construct exceeded the recommended 0.7 thresholds [2]. Convergent validity was also confirmed, as the average variance extracted (AVE) for all items exceeded 0.5 [2]. Discriminant validity was verified using the Heterotrait-Monotrait Ratio (HTMT) with all constructs exceeding the threshold of 0.85, indicating that constructs are distinct, measuring unique aspects of the model [3].

Table 1:
Indicator loadings

	Factor loading (β)	Standard deviation	t-statistics	*P* values	VIF*
General happiness (A) <- Subjective Wellbeing	0.808	0.005	149.413	< 0.001	1.706
General happiness (B) <- Subjective Wellbeing	0.836	0.004	209.515	< 0.001	1.729
Happiness <- Subjective Wellbeing	0.739	0.007	102.012	< 0.001	1.535
Happy over last week <- Subjective Wellbeing	0.738	0.007	111.406	< 0.001	1.4
Depression <- Subjective Illbeing	0.896	0.003	274.537	< 0.001	2.31
Anxiety <- Subjective Illbeing	0.888	0.004	203.065	< 0.001	2.404
Stress <- Subjective Illbeing	0.826	0.006	138.378	< 0.001	1.663
Belief that own life is meaningful (A) <- MOB	0.713	0.008	87.818	< 0.001	1.459
Belief that own life is meaningful (B) <- MOB	0.742	0.007	101.525	< 0.001	1.47
(Reversed) Thought that life was not worth living (A) <- MOB	0.762	0.007	108.932	< 0.001	1.94
(Reversed) Thought that life was not worth living (B) <- MOB	0.771	0.007	116.649	< 0.001	1.941
(Reversed) Frequency of feeling isolated from others <- Social Connectedness	0.876	0.004	217.436	< 0.001	2.453

(Reversed) Frequency of feeling left out <- Social Connectedness	0.852	0.005	184.587	< 0.001	2.161
(Reversed) Frequency of feeling that lacks companionship <- Social Connectedness	0.817	0.006	136.664	< 0.001	1.874
Frequency of feeling in tune with people <- Social Connectedness	0.677	0.01	67.768	< 0.001	1.294
(Reversed) Tendency to take a long time to get over setbacks <- Resilience	0.86	0.005	188.021	< 0.001	2.474
(Reversed) Hard to snap back when something bad happens <- Resilience	0.855	0.005	181.384	< 0.001	2.456
Comes through difficult times with little trouble <- Resilience	0.83	0.006	137.582	< 0.001	2.088
Quick recovery from stressful events <- Resilience	0.782	0.008	93.291	< 0.001	1.883
Tendency to bounce back quickly after hard times <- Resilience	0.670	0.012	56.51	< 0.001	1.441
Felt hated by family member as a child (A) <- Lifetime Adversity	0.781	0.009	87.902	< 0.001	2.251
Felt hated by family member as a child (B) <- Lifetime Adversity	0.800	0.008	104.187	< 0.001	2.319
(Reversed) Felt loved as a child (A) <- Lifetime Adversity	0.768	0.009	90.024	< 0.001	2.316
(Reversed) Felt loved as a child (B) <- Lifetime Adversity	0.798	0.008	104.04	< 0.001	2.438
Physically abused by family as a child (A) <- Lifetime Adversity	0.663	0.015	42.996	< 0.001	2.442
Physically abused by family as a child (B) <- Lifetime Adversity	0.683	0.015	45.236	< 0.001	2.515
QE (MOB) -> QE (MOB)	1	1	0	n/a	1
Townsend deprivation index at recruitment -> Current Adversity	1	0	n/a	n/a	1
RMSSD -> HRV	1	0	n/a	n/a	1
Lifetime Adversity x MOB -> Lifetime Adversity x MOB	1	0	n/a	n/a	1
Social Connectedness x MOB -> Social Connectedness x MOB	1	0	n/a	n/a	1

*VIF: Variance Inflated Factor.

Table 2:
Reliability and convergent validity of constructs

	Cronbach's alpha	Composite reliability (rho_a)	Composite reliability (rho_c)	Average variance extracted (AVE)
Lifetime Adversity	0.849	0.867	0.885	0.564
Resilience	0.861	0.879	0.900	0.644
Social Connectedness	0.820	0.828	0.883	0.655
Subjective Illbeing	0.840	0.846	0.904	0.758
Subjective Wellbeing	0.787	0.797	0.862	0.611
MOB	0.736	0.738	0.835	0.558

Table 3:
HTMT Discriminant validity results

	Heterotrait-monotrait ratio (HTMT)
Resilience <-> Lifetime Adversity	0.188
Social Connectedness <-> Lifetime Adversity	0.322
Social Connectedness <-> Resilience	0.468
Subjective Illbeing <-> Lifetime Adversity	0.361
Subjective Illbeing <-> Resilience	0.618
Subjective Illbeing <-> Social Connectedness	0.643
Subjective Wellbeing <-> Lifetime Adversity	0.287
Subjective Wellbeing <-> Resilience	0.571
Subjective Wellbeing <-> Social Connectedness	0.646
Subjective Wellbeing <-> Subjective Illbeing	0.744
MOB <-> Lifetime Adversity	0.385
MOB <-> Resilience	0.552
MOB <-> Social Connectedness	0.646
MOB <-> Subjective Illbeing	0.765
MOB <-> Subjective Wellbeing	0.811

Structural Model Assessment

To assess the structural model, a bootstrapping procedure was performed on 10,000 samples at a 5% significance level to evaluate the strength and significance of path coefficients, explained variance (R^2), and model fit [4, 5]. Predictive relevance (Q^2) was assessed using the PLS-Predict method [6].

The R-squared values for MOB (0.110), social connectedness (0.289), resilience (0.239), wellbeing (0.518), and illbeing (0.516) indicate that the model explains a significant portion of the variance in these constructs, with all values exceeding the 0.10 threshold [1]. Additionally, the Q-squared values for MOB (0.103), social connectedness (0.079), resilience

(0.031), wellbeing (0.064), and illbeing (0.097) all exceeded zero, supporting the predictive power of the model.

Goodness of fit was evaluated using the Standardised Root Mean Square Residual (SRMR = 0.064) and Normed Fit Index (NFI = 0.778). The SRMR value fell below the 0.08 cut-off, indicating a good fit between the model predictions and the observed data [7]. However, the NFI fell short of the preferred standard of 0.90 [8]. This lower value is attributed to the model's complexity, the improvement over a null model (NFI > 0) validates the theoretical consistency and predictive validity of our model [9]. We also note that as PLS-SEM is inherently prediction-oriented, it prioritises maximising the explained variance (e.g. R^2 and Q^2) over global covariance fit indices (e.g. CFI) typically used in Covariance-Based SEM. When the measurement model demonstrates strong reliability and validity, high R^2 and Q^2 values reliably reflect the model’s capacity to predict outcomes, even if some global fit indices are less than optimal [3, 10]. Together, these predictive measures and fit indices substantiate the adequacy of our model.

Table 4:
Direct path relationships from full dataset PLS-SEM

	β	Standard deviation	*t* statistics	*P* values
Current Adversity -> MOB	-0.086	0.012	7.494	< 0.001
HRV -> MOB	0.024	0.009	2.626	0.004
HRV -> Subjective Wellbeing	0.02	0.008	2.458	0.007
Lifetime Adversity -> Current Adversity	0.088	0.013	6.887	< 0.001
Lifetime Adversity -> MOB	-0.312	0.012	25.717	< 0.001
Lifetime Adversity -> Social Connectedness	-0.132	0.013	10.001	< 0.001
Lifetime Adversity -> Subjective Illbeing	0.072	0.01	7.513	< 0.001
MOB -> Resilience	0.33	0.012	26.453	< 0.001
MOB -> Social Connectedness	0.421	0.012	36.138	< 0.001
MOB -> Subjective Illbeing	-0.296	0.012	23.826	< 0.001
MOB -> Subjective Wellbeing	0.333	0.011	29.391	< 0.001
QE (MOB) -> Social Connectedness	-0.054	0.01	5.569	< 0.001
Resilience -> Subjective Illbeing	-0.281	0.01	27.014	< 0.001

Resilience -> Subjective Wellbeing	0.124	0.01	12.215	< 0.001
Social Connectedness -> Resilience	0.228	0.013	17.871	< 0.001
Social Connectedness -> Subjective Illbeing	-0.159	0.012	12.732	< 0.001
Social Connectedness -> Subjective Wellbeing	0.205	0.012	17.769	< 0.001
Subjective Illbeing -> Subjective Wellbeing	-0.277	0.012	22.569	< 0.001
Social Connectedness x MOB -> Subjective Illbeing	0.096	0.012	8.345	< 0.001
Social Connectedness x MOB -> Subjective Wellbeing	0.062	0.008	7.598	< 0.001
Lifetime Adversity x MOB -> Subjective Illbeing	-0.042	0.012	3.449	< 0.001

Table 5:
Full dataset total indirect effects

	β	Standard deviation	*T* statistics	*P* values
Current Adversity -> Resilience	-0.037	0.005	7.368	p < 0.001
Current Adversity -> Social Connectedness	-0.036	0.005	7.301	p < 0.001
Current Adversity -> Subjective Illbeing	0.042	0.006	7.4	p < 0.001
Current Adversity -> Subjective Wellbeing	-0.052	0.007	7.46	p < 0.001
HRV -> Resilience	0.01	0.004	2.614	0.004
HRV -> Social Connectedness	0.01	0.004	2.617	0.004
HRV -> Subjective Illbeing	-0.012	0.004	2.615	0.004
HRV -> Subjective Wellbeing	0.015	0.006	2.621	0.004
Lifetime Adversity -> MOB	-0.008	0.002	4.992	p < 0.001
Lifetime Adversity -> Resilience	-0.166	0.007	23.417	p < 0.001
Lifetime Adversity -> Social Connectedness	-0.135	0.006	21.097	p < 0.001
Lifetime Adversity -> Subjective Illbeing	0.183	0.007	25.088	p < 0.001
Lifetime Adversity -> Subjective Wellbeing	-0.252	0.009	27.879	p < 0.001
MOB -> Resilience	0.096	0.006	15.779	p < 0.001
MOB -> Subjective Illbeing	-0.187	0.007	26.441	p < 0.001
MOB -> Subjective Wellbeing	0.273	0.008	34.98	p < 0.001
Resilience -> Subjective Wellbeing	0.078	0.004	17.577	p < 0.001
Social Connectedness -> Subjective Illbeing	-0.064	0.004	15.11	p < 0.001
Social Connectedness -> Subjective Wellbeing	0.09	0.005	17.547	p < 0.001
QE (MOB) -> Resilience	-0.012	0.002	5.34	p < 0.001
QE (MOB) -> Subjective Illbeing	0.012	0.002	5.234	p < 0.001
QE (MOB) -> Subjective Wellbeing	-0.016	0.003	5.435	p < 0.001
Social Connectedness x MOB -> Subjective Wellbeing	-0.027	0.004	7.588	p < 0.001
Lifetime Adversity x MOB -> Subjective Wellbeing	0.012	0.004	3.353	p < 0.001

Table 6:
Full dataset total effects

	β	Standard deviation	*T* statistics	*P* values
Current Adversity -> MOB	-0.086	0.012	7.494	p < 0.001

Current Adversity -> Resilience	-0.037	0.005	7.368	p < 0.001
Current Adversity -> Social Connectedness	-0.036	0.005	7.301	p < 0.001
Current Adversity -> Subjective Illbeing	0.042	0.006	7.4	p < 0.001
Current Adversity -> Subjective Wellbeing	-0.052	0.007	7.46	p < 0.001
HRV -> MOB	0.024	0.009	2.626	0.004
HRV -> Resilience	0.01	0.004	2.614	0.004
HRV -> Social Connectedness	0.01	0.004	2.617	0.004
HRV -> Subjective Illbeing	-0.012	0.004	2.615	0.004
HRV -> Subjective Wellbeing	0.035	0.01	3.384	p < 0.001
Lifetime Adversity -> Current Adversity	0.088	0.013	6.887	p < 0.001
Lifetime Adversity -> MOB	-0.32	0.012	26.223	p < 0.001
Lifetime Adversity -> Resilience	-0.166	0.007	23.417	p < 0.001
Lifetime Adversity -> Social Connectedness	-0.264	0.013	19.651	p < 0.001
Lifetime Adversity -> Subjective Illbeing	0.255	0.011	23.396	p < 0.001
Lifetime Adversity -> Subjective Wellbeing	-0.252	0.009	27.879	p < 0.001
MOB -> Resilience	0.426	0.01	41.069	p < 0.001
MOB -> Social Connectedness	0.421	0.012	36.138	p < 0.001
MOB -> Subjective Illbeing	-0.483	0.011	43.099	p < 0.001
MOB -> Subjective Wellbeing	0.606	0.01	61.399	p < 0.001
Resilience -> Subjective Illbeing	-0.281	0.01	27.014	p < 0.001
Resilience -> Subjective Wellbeing	0.202	0.01	20.597	p < 0.001
Social Connectedness -> Resilience	0.228	0.013	17.871	p < 0.001
Social Connectedness -> Subjective Illbeing	-0.223	0.013	17.269	p < 0.001
Social Connectedness -> Subjective Wellbeing	0.295	0.012	25.036	p < 0.001
Subjective Illbeing -> Subjective Wellbeing	-0.277	0.012	22.569	p < 0.001
QE (MOB) -> Resilience	-0.012	0.002	5.34	p < 0.001
QE (MOB) -> Social Connectedness	-0.054	0.01	5.569	p < 0.001
QE (MOB) -> Subjective Illbeing	0.012	0.002	5.234	p < 0.001
QE (MOB) -> Subjective Wellbeing	-0.016	0.003	5.435	p < 0.001
Social Connectedness x MOB -> Subjective Illbeing	0.096	0.012	8.345	p < 0.001
Social Connectedness x MOB -> Subjective Wellbeing	0.036	0.009	3.899	p < 0.001
Lifetime Adversity x MOB -> Subjective Illbeing	-0.042	0.012	3.449	p < 0.001
Lifetime Adversity x MOB -> Subjective Wellbeing	0.012	0.004	3.353	p < 0.001

References

1. Hair Jr JF, Hult GTM, Ringle CM, Sarstedt M, Danks NP, Ray S. Partial least squares structural equation modeling (PLS-SEM) using R: A workbook: Springer Nature; 2021.
2. Henseler J, Hubona G, Ray PA. Using PLS path modeling in new technology research: updated guidelines. Industrial management & data systems. 2016;116(1):2-20. doi: 10.1108/IMDS-09-2015-0382.
3. Henseler J, Ringle CM, Sarstedt M. A new criterion for assessing discriminant validity in variance-based structural equation modeling. Journal of the academy of marketing science. 2015;43:115-35. doi: 10.1007/s11747-014-0403-8.

4. Becker J-M, Cheah J-H, Gholamzade R, Ringle CM, Sarstedt M. PLS-SEM's most wanted guidance. International Journal of Contemporary Hospitality Management. 2023;35(1):321-46. doi: 10.1108/IJCHM-04-2022-0474.
5. Hair JF, Risher JJ, Sarstedt M, Ringle CM. When to use and how to report the results of PLS-SEM. European business review. 2019;31(1):2-24. doi: 10.1108/EBR-11-2018-0203.
6. Shmueli G, Sarstedt M, Hair JF, Cheah J-H, Ting H, Vaithilingam S, et al. Predictive model assessment in PLS-SEM: guidelines for using PLSpredict. European journal of marketing. 2019;53(11):2322-47. doi: 10.1108/EJM-02-2019-0189.
7. Hu Lt, Bentler PM. Cutoff criteria for fit indexes in covariance structure analysis: Conventional criteria versus new alternatives. Structural equation modeling: a multidisciplinary journal. 1999;6(1):1-55. doi: 10.1080/10705519909540118.
8. Bentler PM, Bonett DG. Significance tests and goodness of fit in the analysis of covariance structures. Psychological bulletin. 1980;88(3):588. doi: 10.1037/0033-2909.88.3.588.
9. Kline RB. Principles and practice of structural equation modeling: Guilford publications; 2023.
10. Sarstedt M, Ringle CM, Hair JF. Partial least squares structural equation modeling. Handbook of market research: Springer; 2021. p. 587-632.
