# Supplementary material for: Pathways, predictors and paradoxes of illbeing and wellbeing in older adults: Insights from a UK Biobank study
Source: PLOS Ment Health. 2025 Sep 3;2(9):e0000336. doi: 10.1371/journal.pmen.0000336 (PMC12798268; doi:10.1371/journal.pmen.0000336)
Supplement: S5 File — (S5_File.PDF) [file pmen.0000336.s006.pdf]

Supplementary 5 - Split-half cross-validation

The supplementary results reported here include additional PLS-SEM analyses conducted on two randomly selected subsamples, each comprising approximately 50% of the total sample. These analyses aim to verify the replicability of our findings. The results confirmed that the findings were consistent and replicable across the two subsamples drawn from the entire cohort.

Across both subsamples, the PLS-SEM analysis demonstrated high reliability and convergent validity for all constructs, with Cronbach's alpha and composite reliability values exceeding recommended thresholds. Indicator loadings for each latent variable were consistently high, supporting the robustness of the measurement model. Multicollinearity was not a concern, as indicated by VIF values well below the acceptable limit of 5. Discriminant validity was established through the HTMT ratio, with values below the threshold of 0.85.

Direct path relationships revealed significant effects consistent across both subsamples, reinforcing the hypothesised relationships between constructs. The explained variance for key constructs (R-squared values) was substantial, indicating a strong model fit. Overall, the model fit indices demonstrated a good fit for the data.

The indirect and total effects analyses provided additional support for the robustness of the model, showing consistent mediation effects across both subsamples. These results collectively validate the replicability and robustness of our primary findings.

Figure 4:
PLS-SEM; Subsample 1

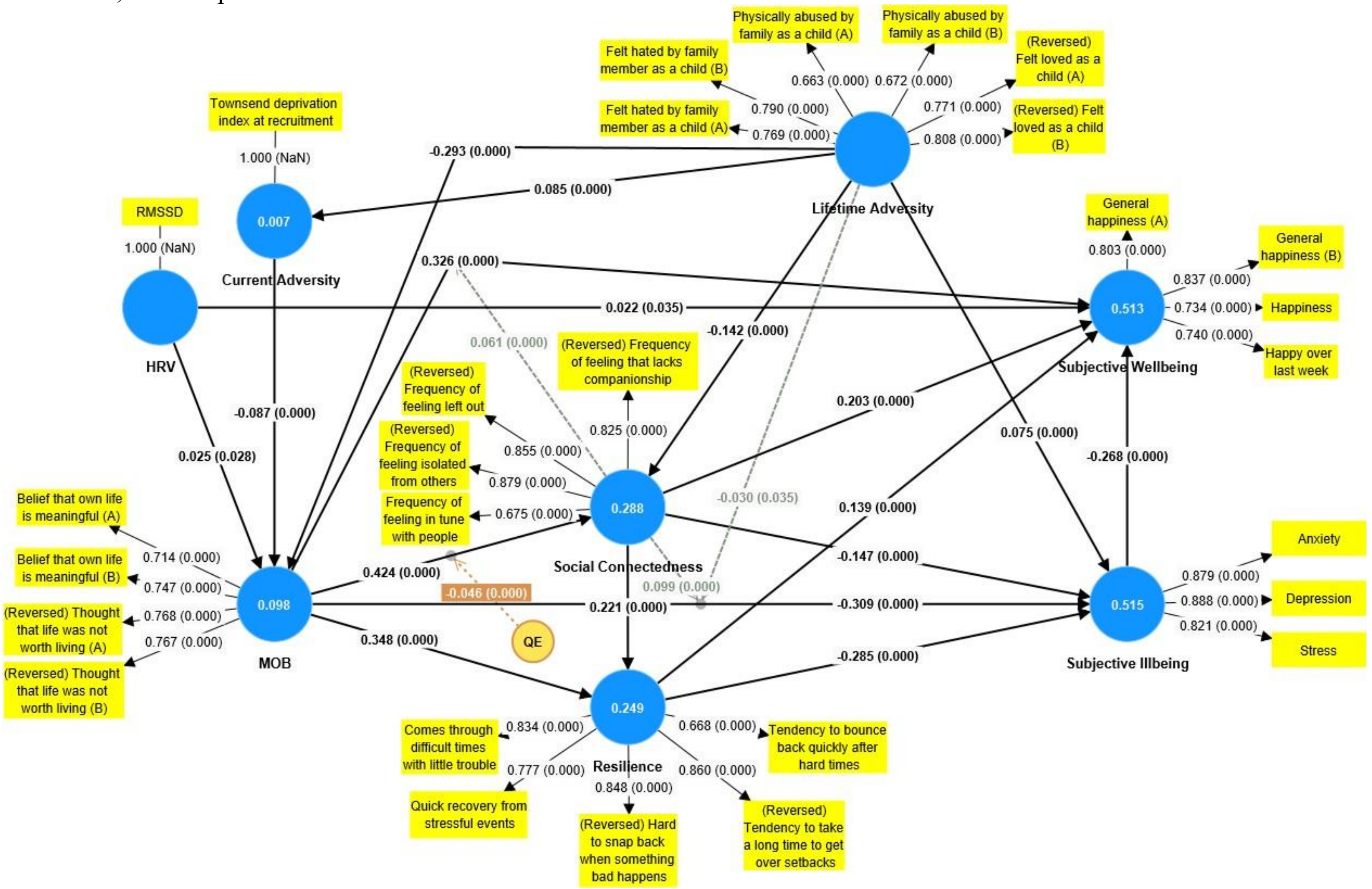

Table 7:
Reliability and convergent validity of constructs (First split)

	Cronbach's alpha	Composite reliability (rho_a)	Composite reliability (rho_c)	Average variance extracted (AVE)
Lifetime Adversity	0.847	0.868	0.883	0.559
MOB	0.739	0.741	0.836	0.561
Resilience	0.858	0.876	0.898	0.641
Social Connectedness	0.824	0.833	0.885	0.66
Subjective Illbeing	0.829	0.836	0.898	0.746
Subjective Wellbeing	0.785	0.797	0.861	0.608

Table 8:
Indicator loadings (First split)

	Factor loading (β)	Standard deviation	*T* statistics	*P* values	VIF
(Reversed) Felt loved as a child (A) <- Lifetime Adversity	0.771	0.013	60.515	$p < 0.001$	2.318
(Reversed) Felt loved as a child (B) <- Lifetime Adversity	0.808	0.011	70.88	$p < 0.001$	2.466
Felt hated by family member as a child (A) <- Lifetime Adversity	0.769	0.013	57.59	$p < 0.001$	2.236
Felt hated by family member as a child (B) <- Lifetime Adversity	0.79	0.011	70.035	$p < 0.001$	2.317
Physically abused by family as a child (A) <- Lifetime Adversity	0.663	0.022	30.676	$p < 0.001$	2.588
Physically abused by family as a child (B) <- Lifetime Adversity	0.672	0.022	31.221	$p < 0.001$	2.63
Belief that own life is meaningful (A) <- MOB	0.714	0.011	62.319	$p < 0.001$	1.489
Belief that own life is meaningful (B) <- MOB	0.747	0.01	74.215	$p < 0.001$	1.504
(Reversed) Thought that life was not worth living (A) <- MOB	0.768	0.009	81.172	$p < 0.001$	1.901
(Reversed) Thought that life was not worth living (B) <- MOB	0.767	0.01	80.009	$p < 0.001$	1.883

(Reversed) Frequency of feeling isolated from others <- Social Connectedness	0.879	0.006	155.665	$p < 0.001$	2.477
(Reversed) Frequency of feeling left out <- Social Connectedness	0.855	0.006	134.114	$p < 0.001$	2.142
(Reversed) Frequency of feeling that lacks companionship <- Social Connectedness	0.825	0.008	103.247	$p < 0.001$	1.95
Frequency of feeling in tune with people <- Social Connectedness	0.675	0.014	47.232	$p < 0.001$	1.309
(Reversed) Hard to snap back when something bad happens <- Resilience	0.848	0.007	123.087	$p < 0.001$	2.374
(Reversed) Tendency to take a long time to get over setbacks <- Resilience	0.86	0.006	133.403	$p < 0.001$	2.464
Comes through difficult times with little trouble <- Resilience	0.834	0.008	99.81	$p < 0.001$	2.132
Quick recovery from stressful events <- Resilience	0.777	0.012	63.739	$p < 0.001$	1.863
Tendency to bounce back quickly after hard times <- Resilience	0.668	0.017	39.434	$p < 0.001$	1.425
General happiness (A) <- Subjective Wellbeing	0.803	0.008	96.691	$p < 0.001$	1.724
General happiness (B) <- Subjective Wellbeing	0.837	0.005	157.515	$p < 0.001$	1.497
Happy over last week <- Subjective Wellbeing	0.74	0.009	80.881	$p < 0.001$	1.419
Happiness <- Subjective Wellbeing	0.734	0.01	73.253	$p < 0.001$	1.497
Anxiety <- Subjective Illbeing	0.879	0.007	133.164	$p < 0.001$	2.236
Depression <- Subjective Illbeing	0.888	0.005	184.748	$p < 0.001$	2.133
Stress <- Subjective Illbeing	0.821	0.009	94.939	$p < 0.001$	1.632
RMSSD -> HRV	1	0	n/a	n/a	1
Townsend deprivation index at recruitment -> Current Adversity	1	0	n/a	n/a	1
QE (MOB) -> QE (MOB)	1	0	n/a	n/a	1
Lifetime Adversity x MOB -> Lifetime Adversity x MOB	1	0	n/a	n/a	1
Social Connectedness x MOB -> Social Connectedness x MOB	1	0	n/a	n/a	1

Table 9:
HTMT Discriminant validity results (First split)

	Heterotrait-monotrait ratio (HTMT)
MOB <-> Lifetime Adversity	0.356
Resilience <-> Lifetime Adversity	0.201
Resilience <-> MOB	0.571
Social Connectedness <-> Lifetime Adversity	0.321
Social Connectedness <-> MOB	0.659
Social Connectedness <-> Resilience	0.47
Subjective Illbeing <-> Lifetime Adversity	0.344
Subjective Illbeing <-> MOB	0.773
Subjective Illbeing <-> Resilience	0.633
Subjective Illbeing <-> Social Connectedness	0.633
Subjective Wellbeing <-> Lifetime Adversity	0.277
Subjective Wellbeing <-> MOB	0.809
Subjective Wellbeing <-> Resilience	0.589
Subjective Wellbeing <-> Social Connectedness	0.637
Subjective Wellbeing <-> Subjective Illbeing	0.746

Table 10:
Direct path relationships from full dataset PLS-SEM (First split)

	β	Standard deviation	*T* statistics	*P* values
Current Adversity -> MOB	-0.087	0.016	5.313	p < 0.001
HRV -> Personal Values	0.025	0.013	1.911	0.028
HRV -> Subjective Wellbeing	0.022	0.012	1.812	0.035
Lifetime Adversity -> Current Adversity	0.085	0.019	4.57	p < 0.001
Lifetime Adversity -> MOB	-0.293	0.017	16.891	p < 0.001
Lifetime Adversity -> Social Connectedness	-0.142	0.018	7.898	p < 0.001
Lifetime Adversity -> Subjective Illbeing	0.075	0.014	5.527	p < 0.001
MOB -> Resilience	0.348	0.017	20.075	p < 0.001
MOB -> Social Connectedness	0.424	0.017	25.044	p < 0.001
MOB -> Subjective Illbeing	-0.309	0.016	18.717	p < 0.001
MOB -> Subjective Wellbeing	0.326	0.016	20.403	p < 0.001
Resilience -> Subjective Illbeing	-0.285	0.015	19.511	p < 0.001
Resilience -> Subjective Wellbeing	0.139	0.014	9.599	p < 0.001
Social Connectedness -> Resilience	0.221	0.017	12.804	p < 0.001
Social Connectedness -> Subjective Illbeing	-0.147	0.016	8.959	p < 0.001
Social Connectedness -> Subjective Wellbeing	0.203	0.016	12.711	p < 0.001
Subjective Illbeing -> Subjective Wellbeing	-0.268	0.016	16.295	p < 0.001
QE (Personal Values) -> Social Connectedness	-0.046	0.014	3.367	p < 0.001
Social Connectedness x MOB -> Subjective Illbeing	0.099	0.016	6.319	p < 0.001

Social Connectedness x MOB -> Subjective Wellbeing	0.061	0.012	4.927	p < 0.001
Lifetime Adversity x MOB -> Subjective Illbeing	-0.030	0.017	1.807	0.035

Table 11:
R2 (First split)

	R-square
Current Adversity	0.007
Resilience	0.249
Social Connectedness	0.285
Subjective Illbeing	0.515
Subjective Wellbeing	0.513
MOB	0.098

Table 12:
Model fit indices (First split)

	Saturated model	Estimated model
SRMR	0.063	0.062
NFI	0.764	0.777

Figure 5:
PLS-SEM; Subsample 2

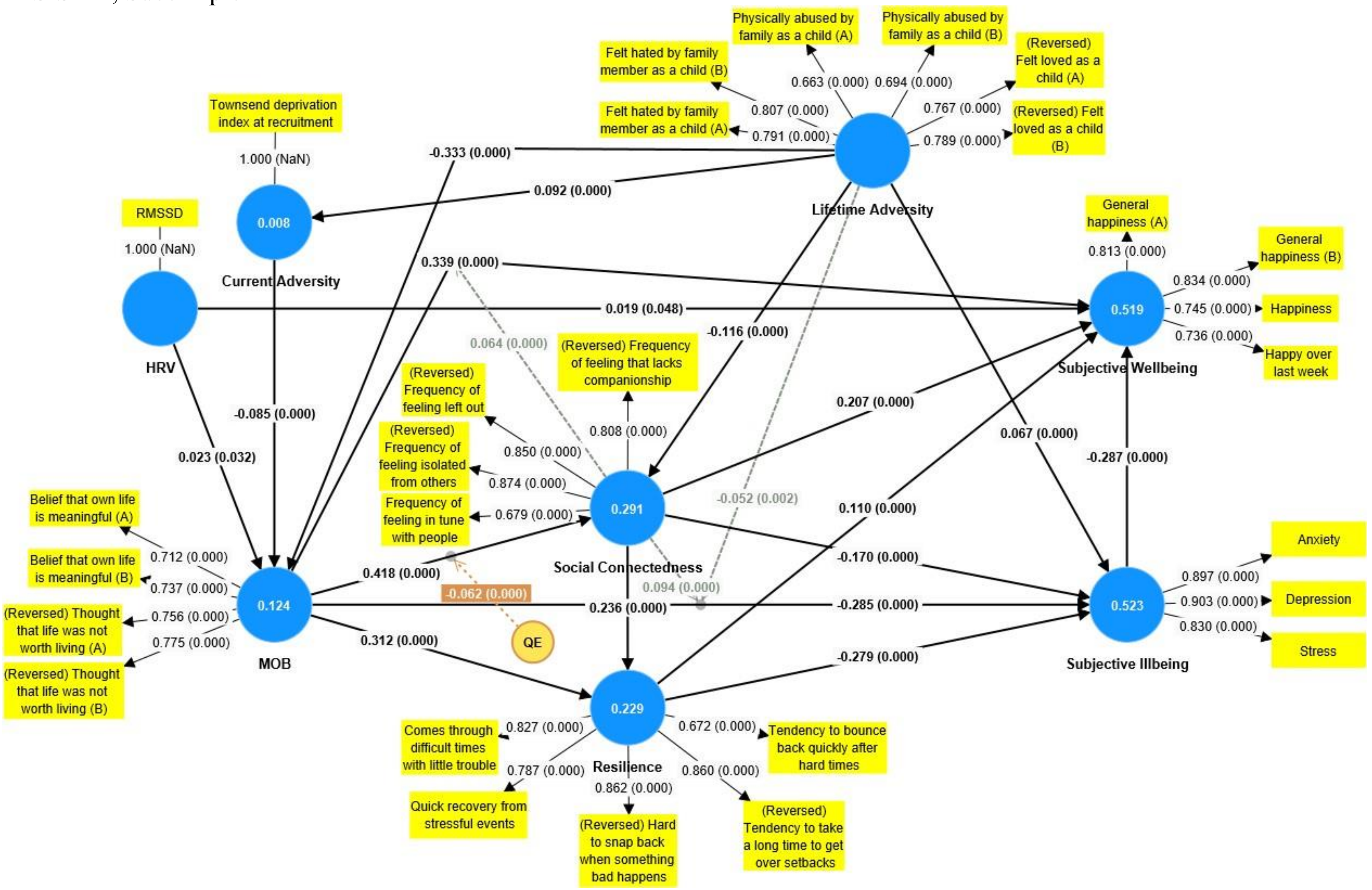

Table 13:
Reliability and convergent validity of constructs (Second split)

	Cronbach's alpha	Composite reliability (rho_a)	Composite reliability (rho_c)	Average variance extracted (AVE)
Lifetime Adversity	0.851	0.868	0.887	0.568
Resilience	0.863	0.882	0.901	0.648
Social Connectedness	0.816	0.822	0.88	0.65
Subjective Illbeing	0.85	0.855	0.909	0.77
Subjective Wellbeing	0.789	0.798	0.863	0.613
MOB	0.733	0.734	0.833	0.555

Table 14:
Indicator loadings (Second split)

	Factor loading (β)	Standard deviation	*T* statistics	*P* values	VIF
(Reversed) Felt loved as a child (A) <- Lifetime Adversity	0.767	0.012	66.601	p < 0.001	2.319
(Reversed) Felt loved as a child (B) <- Lifetime Adversity	0.789	0.01	77.105	p < 0.001	2.413
Felt hated by family member as a child (A) <- Lifetime Adversity	0.791	0.012	66.098	p < 0.001	2.268
Felt hated by family member as a child (B) <- Lifetime Adversity	0.807	0.01	77.196	p < 0.001	2.323
Physically abused by family as a child (A) <- Lifetime Adversity	0.663	0.022	29.905	p < 0.001	2.312
Physically abused by family as a child (B) <- Lifetime Adversity	0.694	0.021	32.855	p < 0.001	2.417
Belief that own life is meaningful (A) <- MOB	0.712	0.011	62.492	p < 0.001	1.431
Belief that own life is meaningful (B) <- MOB	0.737	0.01	70.603	p < 0.001	1.439
(Reversed) Thought that life was not worth living (A) <- MOB	0.756	0.01	76.482	p < 0.001	1.983
(Reversed) Thought that life was not worth living (B) <- MOB	0.775	0.009	85.457	p < 0.001	2.005

(Reversed) Frequency of feeling isolated from others <- Social Connectedness	0.874	0.006	149.631	p < 0.001	2.437
(Reversed) Frequency of feeling left out <- Social Connectedness	0.85	0.007	126.407	p < 0.001	2.186
(Reversed) Frequency of feeling that lacks companionship <- Social Connectedness	0.808	0.009	91.453	p < 0.001	1.81
Frequency of feeling in tune with people <- Social Connectedness	0.679	0.014	48.693	p < 0.001	1.282
(Reversed) Hard to snap back when something bad happens <- Resilience	0.862	0.006	134.744	p < 0.001	2.557
(Reversed) Tendency to take a long time to get over setbacks <- Resilience	0.86	0.006	132.42	p < 0.001	2.489
Comes through difficult times with little trouble <- Resilience	0.827	0.009	93.876	p < 0.001	2.049
Quick recovery from stressful events <- Resilience	0.787	0.012	68.22	p < 0.001	1.915
Tendency to bounce back quickly after hard times <- Resilience	0.672	0.017	39.98	p < 0.001	1.459
General happiness (A) <- Subjective Wellbeing	0.813	0.007	117.701	p < 0.001	1.754
General happiness (B) <- Subjective Wellbeing	0.834	0.006	143.816	p < 0.001	1.735
Happy over last week <- Subjective Wellbeing	0.736	0.01	76.732	p < 0.001	1.383
Happiness <- Subjective Wellbeing	0.745	0.011	70.405	p < 0.001	1.383
Anxiety <- Subjective Illbeing	0.897	0.006	157.981	p < 0.001	2.598
Depression <- Subjective Illbeing	0.903	0.004	208.664	p < 0.001	2.516
Stress <- Subjective Illbeing	0.83	0.008	101.415	p < 0.001	1.697
RMSSD -> HRV	1	0	n/a	n/a	1
Townsend deprivation index at recruitment -> Current Adversity	1	0	n/a	n/a	1
QE (MOB) -> QE (MOB)	1	0	n/a	n/a	1
Social Connectedness x MOB -> Social Connectedness x MOB	1	0	n/a	n/a	1
Lifetime Adversity x MOB -> Lifetime Adversity x MOB	1	0	n/a	n/a	1

Table 15:
HTMT Discriminant validity results (Second split)

	Heterotrait-monotrait ratio (HTMT)
Resilience <-> Lifetime Adversity	0.175
Social Connectedness <-> Lifetime Adversity	0.324
Social Connectedness <-> Resilience	0.466
Subjective Illbeing <-> Lifetime Adversity	0.376
Subjective Illbeing <-> Resilience	0.604
Subjective Illbeing <-> Social Connectedness	0.653
Subjective Wellbeing <-> Lifetime Adversity	0.298
Subjective Wellbeing <-> Resilience	0.553
Subjective Wellbeing <-> Social Connectedness	0.655
Subjective Wellbeing <-> Subjective Illbeing	0.742
MOB <-> Lifetime Adversity	0.414
MOB <-> Resilience	0.534
MOB <-> Social Connectedness	0.669
MOB <-> Subjective Illbeing	0.758
MOB <-> Subjective Wellbeing	0.814

Table 16:
Direct path relationships from full dataset PLS-SEM (Second split)

	β	Standard deviation	*T* statistics	*P* values
Current Adversity -> MOB	-0.085	0.016	5.369	p < 0.001
HRV -> MOB	0.023	0.012	1.848	0.032
HRV -> Subjective Wellbeing	0.019	0.012	1.661	0.048
Lifetime Adversity -> Current Adversity	0.092	0.018	5.178	p < 0.001
Lifetime Adversity -> MOB	-0.333	0.017	19.885	p < 0.001
Lifetime Adversity -> Social Connectedness	-0.116	0.019	6.074	p < 0.001
Lifetime Adversity -> Subjective Illbeing	0.067	0.013	5.031	p < 0.001
MOB -> Resilience	0.312	0.018	17.687	p < 0.001
MOB -> Social Connectedness	0.418	0.016	26.788	p < 0.001
MOB -> Subjective Illbeing	-0.285	0.018	15.426	p < 0.001
MOB -> Subjective Wellbeing	0.339	0.016	21.383	p < 0.001
Resilience -> Subjective Illbeing	-0.279	0.015	19.196	p < 0.001
Resilience -> Subjective Wellbeing	0.11	0.014	7.795	p < 0.001

Social Connectedness -> Resilience	0.236	0.018	12.96	p < 0.001
Social Connectedness -> Subjective Illbeing	-0.17	0.018	9.208	p < 0.001
Social Connectedness -> Subjective Wellbeing	0.207	0.016	12.961	p < 0.001
Subjective Illbeing -> Subjective Wellbeing	-0.287	0.018	15.622	p < 0.001
QE (MOB) -> Social Connectedness	-0.062	0.014	4.489	p < 0.001
Social Connectedness x MOB -> Subjective Illbeing	0.094	0.017	5.625	p < 0.001
Social Connectedness x MOB -> Subjective Wellbeing	0.064	0.011	5.831	p < 0.001
Lifetime Adversity x MOB -> Subjective Illbeing	-0.052	0.018	2.914	0.002

Table 17:
R2 (Second split)

	R-square
Current Adversity	0.008
Resilience	0.229
Social Connectedness	0.284
Subjective Illbeing	0.523
Subjective Wellbeing	0.519
MOB	0.124

Table 18:
Model fit indices (Second split)

	Saturated model	Estimated model
SRMR	0.062	0.064
NFI	0.774	0.778
