# Supplementary material for: Pathways, predictors and paradoxes of illbeing and wellbeing in older adults: Insights from a UK Biobank study
Source: PLOS Ment Health. 2025 Sep 3;2(9):e0000336. doi: 10.1371/journal.pmen.0000336 (PMC12798268; doi:10.1371/journal.pmen.0000336)
Supplement: S6 File — (S6_File.PDF) [file pmen.0000336.s007.pdf]

Supplementary 6 - Predictive Performance analysis (PLS-Predict)

To validate the predictive performance of our PLS-SEM, we employed the PLS-Predict method [1] on the full dataset, utilising 10-fold cross-validation. The PLS-Predict metric of interest was Q^2, in which values above 0 indicate that the model has significant predictive power for the endogenous constructs.

Table 19:
PLS-Predict results

	Q^2
Current Adversity (Area)	0.007
Resilience	0.031
Social Connectedness	0.081
Subjective Illbeing	0.097
Subjective Wellbeing	0.067
Values	0.103

References

1. Shmueli G, Sarstedt M, Hair JF, Cheah J-H, Ting H, Vaithilingam S, et al. Predictive model assessment in PLS-SEM: guidelines for using PLSpredict. European journal of marketing. 2019;53(11):2322-47. doi: 10.1108/EJM-02-2019-0189.
