# Supplementary material for: Pathways, predictors and paradoxes of illbeing and wellbeing in older adults: Insights from a UK Biobank study
Source: PLOS Ment Health. 2025 Sep 3;2(9):e0000336. doi: 10.1371/journal.pmen.0000336 (PMC12798268; doi:10.1371/journal.pmen.0000336)
Supplement: S7 File — (S7_File.PDF) [file pmen.0000336.s008.pdf]

Supplementary 7 - Network Centrality Analysis results

Table 20:
Network Centrality Analysis results

Variable	Degree Centrality	Closeness Centrality	Betweenness Centrality
Current Adversity	0.22	0.11	0
MOB	0.78	0.33	0.11
HRV	0.22	0	0
Subjective Wellbeing	0.67	0.75	0
Lifetime Adversity	0.44	0	0
Social Connectedness	0.56	0.30	0.01
Subjective Illbeing	0.78	0.71	0.02
Resilience	0.44	0.35	0
Social Connectedness x MOB	0.22	0	0
Lifetime Adversity x MOB	0.11	0	0
