# Supplementary material for: Pathways, predictors and paradoxes of illbeing and wellbeing in older adults: Insights from a UK Biobank study
Source: PLOS Ment Health. 2025 Sep 3;2(9):e0000336. doi: 10.1371/journal.pmen.0000336 (PMC12798268; doi:10.1371/journal.pmen.0000336)
Supplement: S8 File — (S8_File.PDF) [file pmen.0000336.s009.pdf]

**Supplementary 8 - Function Comparison**

We investigated several model structures within a Bayesian regression framework, to understand the predictive relationships between subjective wellbeing and illbeing. The models considered included either linear, quadratic, cubic, sigmoid, exponential, or logarithmic terms. To compare model fits and determine the best fitting model, we used the Watanabe-Akaike Information Criterion (WAIC) [1] and Leave-One-Out cross-validation (LOO) [2]. Models with lower WAIC and LOO scores indicate better fit, reflecting higher predictive accuracy and appropriate penalisation for complexity [2].

**Table 21:**
Model fit scores for factors predicting wellbeing

| Wellbeing | Linear | | Quadratic | | Sigmoid | | Cubic | | Exponential | | Logarithmic | |
|---|---|---|---|---|---|---|---|---|---|---|---|---|
| | WAIC | LOO | WAIC | LOO | WAIC | LOO | WAIC | LOO | WAIC | LOO | WAIC | LOO |
| HRV | -659.28 | -659.28 | -657.86 | -657.86 | -659.20 | -659.20 | -658.83 | -658.83 | -657.26 | -657.26 | -659.18 | -659.18 |
| Values | 155.93 | 155.92 | 73.83 | 73.83 | 157.99 | 157.99 | -43.69 | -43.69 | 126.70 | 126.70 | 156.06 | 156.05 |
| Social Connectedness | -230.49 | -230.49 | -271.13 | -271.13 | -226.06 | -226.07 | -286.83 | -286.84 | -250.04 | -250.04 | -216.23 | -216.23 |
| Resilience | -279.22 | -279.23 | -330.66 | -330.67 | -276.96 | -276.97 | -376.43 | -376.43 | -298.65 | -298.66 | -271.54 | -271.55 |
| Illbeing | 65.55 | 65.55 | 106.21 | 106.20 | 53.05 | 53.05 | 79.92 | 79.91 | 101.98 | 101.98 | 25.51 | 25.51 |

**Table 22:**
Model fit scores for factors predicting illbeing

| Illbeing | Linear | | Quadratic | | Sigmoid | | Cubic | | Exponential | | Logarithmic | |
|---|---|---|---|---|---|---|---|---|---|---|---|---|
| | WAIC | LOO | WAIC | LOO | WAIC | LOO | WAIC | LOO | WAIC | LOO | WAIC | LOO |
| Lifetime Adversity | -470.99 | -470.99 | -433.69 | -433.69 | -474.06 | -474.06 | -432.36 | -432.36 | -457.24 | -457.24 | -479.95 | -479.95 |
| Values | -211.71 | -211.71 | -282.54 | -282.55 | -205.37 | -205.38 | -349.41 | -349.41 | -244.38 | -244.39 | -197.19 | -197.20 |
| Social Connectedness | -196.04 | -196.04 | -237.53 | -237.53 | -191.38 | -191.39 | -252.51 | -252.51 | -216.13 | -216.14 | -180.40 | -180.40 |
| Resilience | 5.03 | 5.02 | -107.78 | -107.79 | 13.50 | 13.49 | -197.55 | -197.56 | -44.39 | -44.39 | 28.95 | 28.94 |

**References**

1. Watanabe S, Opper M. Asymptotic equivalence of Bayes cross validation and widely applicable information criterion in singular learning theory. Journal of machine learning research. 2010;11(12).
2. Vehtari A, Gelman A, Gabry J. Practical Bayesian model evaluation using leave-one-out cross-validation and WAIC. Statistics and computing. 2017;27:1413-32. doi: 10.1007/s11222-016-9696-4.
