# Supplementary material for: Pathways, predictors and paradoxes of illbeing and wellbeing in older adults: Insights from a UK Biobank study
Source: PLOS Ment Health. 2025 Sep 3;2(9):e0000336. doi: 10.1371/journal.pmen.0000336 (PMC12798268; doi:10.1371/journal.pmen.0000336)
Supplement: S9 File — (S9_File.PDF) [file pmen.0000336.s010.pdf]

Supplementary 9 - Bayesian Regression Model Diagnostics and Predictive Validation

The final integrated Bayesian regression model demonstrated strong convergence and reliability across all parameters. R-hat values were equal to 1.00 for every estimated parameter, indicating that the two MCMC chains converged successfully. ESS were consistently high across both bulk and tail estimates, exceeding recommended thresholds for stability. For example, bulk ESS values ranged from 1,754 (Subjective Illbeing intercept) to 3,626 (Wellbeing residual variance), and tail ESS values ranged from 1,278 to 1,606, confirming that the posterior distributions were well explored and robustly estimated.

To assess practical significance, we applied the ROPE criterion. All primary predictors had less than 5% of their posterior distributions within the ROPE interval around zero, indicating that their effects were not only statistically credible but also practically meaningful. Specifically, ROPE percentages were 0% for MOB, social connectedness, resilience, illbeing, and lifetime adversity, and 3.35% for HRV, suggesting borderline practical relevance for the physiological predictor.

The model's predictive performance was evaluated using the held-out test dataset ($n = 4,023$). Predictive accuracy was quantified using Bayesian R^2, which reflects the proportion of variance in each outcome explained by the model. The Bayesian R^2 for Subjective Wellbeing was 0.404, while the Bayesian R^2 for Illbeing was 0.543, indicating moderate to strong predictive power. On the 0–1 scale, the model also achieved low prediction error, the mean absolute error (MAE) was 0.162 for wellbeing and 0.179 for illbeing, and the root mean square error (RMSE) was 0.203 and 0.220, respectively. These values confirm that the model's predictions were closely aligned with the observed data.
