# Supplementary material for: Pathways, predictors and paradoxes of illbeing and wellbeing in older adults: Insights from a UK Biobank study
Source: PLOS Ment Health. 2025 Sep 3;2(9):e0000336. doi: 10.1371/journal.pmen.0000336 (PMC12798268; doi:10.1371/journal.pmen.0000336)
Supplement: S10 File — (S10_File.PDF) [file pmen.0000336.s011.pdf]

Supplementary 10 – PLS-SEM Internal Validity and Multigroup Analysis

Permutation-based multigroup analysis across age, sex, and ethnicity

We conducted permutation-based multigroup analyses (PMGA) using SmartPLS 4. Prior to testing path differences, we assessed measurement invariance using the MICOM procedure. This evaluates whether latent constructs are measured equivalently across groups. Step 1 assumes configural invariance, while Step 2 uses permutation tests (5,000 samples) to establish compositional invariance ($p > .05$). Step 3 assesses latent mean and variance differences. Where Step 2 confirmed invariance, structural paths were compared using MGA, with differences interpreted as significant at $p < .05$.

Once compositional invariance was established, we used PMGA (5,000 samples) to test whether structural path coefficients significantly differed across groups. Paths with $p < .05$ were interpreted as meaningfully different, while all others were considered invariant.

Age

For the age comparison (youngest quartile: 40–50 years, $n = 2{,}011$; oldest quartile: 62–70 years, $n = 2{,}013$), MICOM confirmed compositional invariance across all constructs, allowing valid comparison of structural paths. Six paths showed significant differences. Lifetime Adversity predicted Current Adversity more strongly in the younger group ($\beta = .111$ vs .020, $p = .006$), while HRV was more strongly linked to Meaning-Oriented Behaviour (MOB) in the older group ($\beta = -.003$ vs .042, $p = .044$). MOB more strongly predicted Resilience in younger adults ($\beta = .367$ vs .306, $p = .040$), and Resilience more strongly predicted lower Illbeing ($\beta = -.298$ vs –.246, $p = .041$). A quadratic effect of MOB on Social Connectedness was also more pronounced in the younger group ($\Delta\beta = -.049$, $p = .034$), and Subjective Illbeing more strongly predicted lower Subjective Wellbeing in this group ($\beta = –$

.553 vs –.486, *p* = .032). All other paths were invariant (*p* > .10), indicating general model stability across age extremes.

Table 24:
MICOM Measurement Invariance Results for Age Groups (Q1: 40–50 years; Q4: 62–70 years)

	Composite correlation	Step 2 p-value	Compositional invariance?	Latent-mean diff. p (Step 3a)	Latent-variance diff. p (Step 3b)
MOB	0.998	0.145	Yes	0.028	0.02
Resilience	0.997	0.117	Yes	0.011	0.017
Social Connectedness	0.998	0.142	Yes	0.02	0.024
Subjective Wellbeing	0.999	0.131	Yes	0.019	0.016
Subjective Illbeing	0.998	0.119	Yes	0.017	0.018
Lifetime Adversity	0.997	0.124	Yes	0.03	0.015

Table 25:
Structural Path Differences by Age Group – Permutation-Based Multigroup Analysis

Structural Path	Beta (Youngest Q, 40-50)	Beta (Oldest Q, 62-70)	Delta Beta	Permutation p-value	Stronger Path
HRV -> MOB	-0.003	0.042	-0.046	0.044	Oldest
Lifetime Adversity -> Current Adversity	0.111	0.02	0.091	0.006	Youngest
MOB -> Resilience	0.367	0.306	0.061	0.04	Youngest
Quadratic Effect of MOB -> Social Connectedness	-0.061	-0.012	-0.049	0.034	Youngest
Resilience -> Subjective Illbeing	-0.298	-0.246	-0.051	0.041	Youngest
Subjective Illbeing -> Subjective Wellbeing	-0.553	-0.486	-0.067	0.032	Youngest

Sex

For the sex comparison (female: $n = 4{,}288$; male: $n = 3{,}760$), compositional invariance was again confirmed. Two structural paths differed significantly between groups. Lifetime Adversity was more strongly associated with Subjective Illbeing in males than females ($\beta = .088$ vs .054, $p = .036$), while the (reverse-scored) association between Social Connectedness and feeling left out was marginally stronger in females ($\beta = .863$ vs .840, $p = .008$). All other paths were invariant ($p > .05$), indicating consistent model structure across sexes.

Table 26:
MICOM Measurement Invariance Results for Sex (Female vs Male)

Construct	Composite correlation	Step 2 p-value	Compositional invariance?	Latent-mean diff. p (Step 3a)	Latent-variance diff. p (Step 3b)
MOB	0.998	0.145	Yes	0.028	0.02
Resilience	0.997	0.117	Yes	0.011	0.017
Social Connectedness	0.998	0.142	Yes	0.02	0.024
Subjective Wellbeing	0.999	0.131	Yes	0.019	0.016
Subjective Illbeing	0.998	0.119	Yes	0.017	0.018
Lifetime Adversity	0.997	0.124	Yes	0.03	0.015

Table 27:
Structural Path Differences by Sex – Permutation-Based Multigroup Analysis

Structural Path	Beta (Female)	Beta (Male)	Delta Beta	Permutation p-value	Stronger Path
Lifetime Adversity -> Subjective Illbeing	0.054	0.088	-0.035	0.036	Male
(Reversed) Feeling Left Out <- Social Connectedness	0.863	0.84	0.023	0.008	Female

Ethnicity

In the ethnicity comparison (White British: *n* = 7,296; Ethnic Minority: *n* = 751), MICOM confirmed compositional invariance for all constructs. Five structural paths differed significantly. HRV showed stronger associations with four downstream outcomes in the Ethnic Minority group: MOB (β = –.044 vs .030, *p* = .014), Resilience (β = –.018 vs .013, *p* = .015), Social Connectedness (β = –.018 vs .012, *p* = .016), and Subjective Illbeing (β = .020 vs –.014, *p* = .015). In contrast, Social Connectedness more strongly predicted Resilience in the White British group (β = .236 vs .149, *p* = .026). All remaining paths were invariant, suggesting overall model generalisability across ethnic groups, though with a potentially more prominent role for HRV-linked processes in Ethnic Minority participants.

Table 28:
MICOM Measurement Invariance Results for Ethnicity (White British vs Ethnic Minority)

Construct	Composite correlation	Step 2 p-value	Compositional invariance?	Latent-mean diff. p (Step 3a)	Latent-variance diff. p (Step 3b)
MOB	0.998	0.137	Yes	0.022	0.019
Resilience	0.997	0.128	Yes	0.018	0.017
Social Connectedness	0.998	0.141	Yes	0.023	0.02
Subjective Wellbeing	0.999	0.139	Yes	0.021	0.018
Subjective Illbeing	0.998	0.135	Yes	0.02	0.017
Lifetime Adversity	0.997	0.125	Yes	0.024	0.016

Table 29:
Structural Path Differences by Ethnicity – Permutation-Based Multigroup Analysis

Structural Path	Beta (White British)	Beta (Ethnic Minority)	Delta Beta	Permutation p-value	Stronger Path
HRV -> MOB	0.03	-0.044	0.073	0.014	Ethnic Minority
Social Connectedness -> Resilience	0.236	0.149	0.088	0.026	White British

HRV -> Resilience	0.013	-0.018	0.031	0.015	Ethnic Minority
HRV -> Social Connectedness	0.012	-0.018	0.03	0.016	Ethnic Minority
HRV -> Subjective Illbeing	-0.014	0.02	-0.035	0.015	Ethnic Minority
Lifetime Adversity -> Resilience	-0.17	-0.132	-0.038	0.043	White British
HRV -> MOB	0.03	-0.044	0.073	0.014	Ethnic Minority
HRV -> Resilience	0.013	-0.018	0.031	0.015	Ethnic Minority
HRV -> Social Connectedness	0.012	-0.018	0.03	0.016	Ethnic Minority
HRV -> Subjective Illbeing	-0.014	0.02	-0.035	0.015	Ethnic Minority
Lifetime Adversity -> Resilience	-0.17	-0.132	-0.038	0.043	White British
Social Connectedness -> Resilience	0.236	0.149	0.088	0.026	White British
HRV -> MOB	0.03	-0.044	0.073	0.014	Ethnic Minority
HRV -> Resilience	0.013	-0.018	0.031	0.015	Ethnic Minority
HRV -> Social Connectedness	0.012	-0.018	0.03	0.016	Ethnic Minority
HRV -> Subjective Illbeing	-0.014	0.02	-0.035	0.015	Ethnic Minority
Lifetime Adversity -> Resilience	-0.17	-0.132	-0.038	0.043	White British
Social Connectedness -> Resilience	0.236	0.149	0.088	0.026	White British
RMSSD -> HRV	1	1	0	0.022	Ethnic Minority
